# Compatible solutes determine the heat resistance of conidia

**DOI:** 10.1186/s40694-023-00168-9

**Published:** 2023-11-13

**Authors:** Sjoerd J. Seekles, Tom van den Brule, Maarten Punt, Jan Dijksterhuis, Mark Arentshorst, Maryam Ijadpanahsaravi, Winfried Roseboom, Gwendolin Meuken, Véronique Ongenae, Jordy Zwerus, Robin A. Ohm, Gertjan Kramer, Han A. B. Wösten, Johannes H. de Winde, Arthur F. J. Ram

**Affiliations:** 1 TiFN, P.O. Box 557, 6700 AN Wageningen, the Netherlands; 2 Institute of Biology Leiden, Microbial Sciences, Leiden University, Sylviusweg 72, 2333 BE Leiden, the Netherlands; 3 Department of Biology, Utrecht University, Padualaan 8, 3584 CH Utrecht, the Netherlands; 4 Westerdijk Fungal Biodiversity Institute, Uppsalalaan 8, 3584 CT Utrecht, the Netherlands; 5 Mass Spectrometry of Biomolecules, Swammerdam Institute for Life Sciences, University of Amsterdam, Science Park 904, 1090 GE Amsterdam, the Netherlands

**Keywords:** Food preservation, Fungal spores, Compatible solutes, Trehalose, Mannitol, Heat shock proteins, *Aspergillus niger*, Heat stress, Transcriptomics, Proteomics

## Abstract

**Background:**

Asexually developed fungal spores (conidia) are key for the massive proliferation and dispersal of filamentous fungi. Germination of conidia and subsequent formation of a mycelium network give rise to many societal problems related to human and animal fungal diseases, post-harvest food spoilage, loss of harvest caused by plant-pathogenic fungi and moulding of buildings. Conidia are highly stress resistant compared to the vegetative mycelium and therefore even more difficult to tackle.

**Results:**

In this study, complementary approaches are used to show that accumulation of mannitol and trehalose as the main compatible solutes during spore maturation is a key factor for heat resistance of conidia. Compatible solute concentrations increase during conidia maturation, correlating with increased heat resistance of mature conidia. This maturation only occurs when conidia are attached to the conidiophore. Moreover, conidia of a mutant *Aspergillus niger* strain, constructed by deleting genes involved in mannitol and trehalose synthesis and consequently containing low concentrations of these compatible solutes, exhibit a sixteen orders of magnitude more sensitive heat shock phenotype compared to wild-type conidia. Cultivation at elevated temperature results in adaptation of conidia with increased heat resistance. Transcriptomic and proteomic analyses revealed two putative heat shock proteins to be upregulated under these conditions. However, conidia of knock-out strains lacking these putative heat shock proteins did not show a reduced heat resistance.

**Conclusions:**

Heat stress resistance of fungal conidia is mainly determined by the compatible solute composition established during conidia maturation. To prevent heat resistant fungal spore contaminants, food processing protocols should consider environmental conditions stimulating compatible solute accumulation and potentially use compatible solute biosynthesis as a novel food preservation target.

**Supplementary Information:**

The online version contains supplementary material available at 10.1186/s40694-023-00168-9.

## Introduction

Filamentous fungi are among the most common food spoilage microbes found across food sectors, including highly processed food products [1]. Airborne asexual fungal spores (conidia) are produced in large numbers by many food-spoilage fungi. Virtually every cubic meter of air contains fungal conidia in varying densities that can reach into hundreds or more [2–5]. Fungal food spoilage is seen in many food sectors, such as in the production of animal feed, dairy products and fruit juices [6–9]. Thermal treatment is among the most commonly used food preservation techniques [10]. However, certain fungi resist the high temperatures due to highly resistant fungal conidia [11]. Recently, highly resistant conidia were reported in *Paecilomyces variotii* DTO 217-A2 surviving up to 22 min at 60 °C, which is within the margins of common milk thermization protocols [12, 13].

**Fig. 1:**
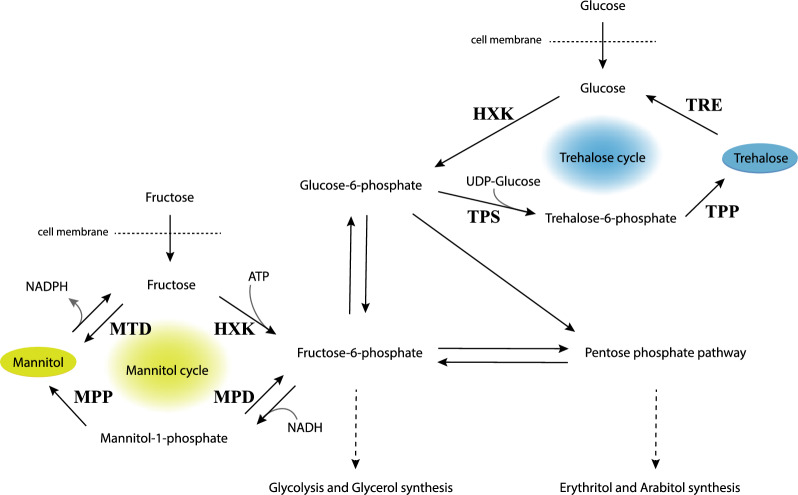
Mannitol and trehalose cycles in aspergilli. Biosynthesis of trehalose in aspergilli, as depicted in blue, starts with the phosphorylation of glucose to glucose-6-phosphate by hexokinase activity (HXK). Glucose-6-phosphate is converted to trehalose-6-phosphate by trehalose-6-phosphate synthase (TPS). Trehalose-6-phosphate phosphatase (TPP) can dephosphorylate trehalose-6-phosphate, yielding trehalose. The catabolism of trehalose is catalyzed by trehalase (TRE) yielding glucose. As represented by the yellow mannitol cycle, mannitol in aspergilli is mainly synthesized from fructose, which is converted to fructose-6-phosphate by HXK activity. Subsequently, fructose-6-phosphate may be reduced to mannitol-1-phosphate by mannitol-1-phosphate dehydrogenase (MPD). Finally, the dephosphorylation of mannitol-1-phosphate is catalyzed by mannitol-1-phosphate phosphatase (MPP), thereby yielding mannitol. The catabolic conversion of mannitol to fructose by mannitol dehydrogenase (MTD) completes the cycle. Both cycles are connected to glycolysis. Additionally, there is a connection with the pentose phosphate pathway and subsequently erythritol and arabitol synthesis. This figure was adapted from figures shown by Svanström et al. and Ruijter et al. [17, 19]

An important parameter determining stress resistance of fungal cells is the amount and composition of internal compatible solutes [14, 15]. These compounds can be quickly accumulated and degraded in the cytoplasm of vegetative cells in response to stress conditions. Compatible solutes like glycerol, erythritol and arabitol are suggested to mainly protect fungal cells against osmotic stress, whereas trehalose and mannitol are implicated to be important for protection against cold, drought and heat stress [15, 16]. The biosynthesis routes of trehalose and mannitol in aspergilli are tightly linked to glycolysis and have been largely described ([17, 18]; Fig. 1).

The role of compatible solutes in vegetative fungal cells is well-documented [20]. Vegetative cells quickly accumulate compatible solutes when exposed to environmental stressors as a quick protective response [21]. However, it is less clear why dormant fungal conidia accumulate compatible solutes to high concentrations. The individual roles of mannitol or trehalose in stress resistance of conidia of *A. niger* have been studied previously [17, 19, 22–25]. Trehalose is responsible for approximately 4–5% of the conidial dry weight of *A. niger* conidia [22]. Three trehalose-6-phosphate synthase genes (*tpsABC*) and three trehalose-6-phosphate phosphatase genes (*tppABC*) have been identified in *A. niger* [19]. For the catabolism of trehalose during germination, the *treB* gene was identified, encoding a neutral trehalase [22]. Additionally, *treA* encodes an acid trehalase, presumably involved in the extracellular degradation of trehalose during vegetative growth [26, 27]. Deletion of the *tpsA* gene in *A. niger* leads to a 56% reduction of the mycelial trehalose content, compared to wild type [23]. In addition, *A. niger* Δ*tpsA* conidia show reduced heat tolerance and a 50% decrease in conidial trehalose content, whereas *tpsB* and *tpsC* deletions showed less effect on both the trehalose content and heat resistance [19].

In addition to trehalose, mannitol provides resistance of *A. niger* conidia to different environmental stressors, including heat [17]. Mannitol is the predominant compatible solute present in *A. niger* conidia and responsible for about 10–15% of the conidial dry weight [24]. Two enzymes are known to impact mannitol concentrations. The mannitol dehydrogenase gene A (*mtdA)* is suggested to be responsible for the mannitol dehydrogenase activity in *A. niger* [25]. The main enzyme for mannitol biosynthesis, however, is the mannitol-6-phosphate dehydrogenase (MPD) enzyme encoded by *mpdA* [17]. In the *A. niger* Δ*mpdA* strain, only 30% of the original mannitol concentration is present inside conidia. Furthermore, Δ*mpdA* conidia show increased sensitivity to stressors like heat and oxidative stress [17].

Other protective molecules, such as heat shock proteins (HSPs) and dehydrins, which may impact conidial stress resistance, accumulate inside conidia of *A. niger* and *A. fumigatus* as confirmed in transcriptomic and proteomic studies [26, 28–30]. Indeed, expression of heat shock proteins is induced in conidia when heat treated for 4 h during conidiation, suggesting that these protective proteins play a role in the increased heat resistance of conidia as an adaptive response to the shift towards a high temperature environment [31]. However, not much is known about their function inside conidia and whether these proteins contribute to the dormant spore’s heat stress resistance.

Here we describe the impact of compatible solute composition and protective proteins on the heat resistance of conidia using *A. niger* as a model organism. We show that young conidia have low heat resistance and contain low concentrations of compatible solutes, both gradually increased during spore maturation. Interestingly, young wild-type conidia as well as mature conidia from a Δ*mpdA,* Δ*tpsABC* knock-out strain lacked most compatible solutes and were both several orders of magnitude more sensitive to heat stress than wild-type conidia. Transcriptome and proteome analyses of conidia revealed upregulation of candidate small protective proteins upon incubation at elevated temperatures, but conidia from knock-out strains lacking these proteins did not show altered heat resistance. Therefore, the compatible solute composition of conidia, accumulated during spore maturation, is the main determinant of their heat resistance.

## Results

### Heat resistance of *A. niger* conidia increases during conidiophore maturation

Previous reports have focussed on the comparison between conidia with age differences of 3 to 8 days [32–34]. However, most conidia are formed during the first three days following inoculation after which many spore chains reach their maximum capacity of approximately 20 conidia per chain [33]. Therefore, we decided to study conidial development during the first three days after inoculation. We compared a time series of conidia harvested from plates as soon as the first conidia were visible (38 h) up to 72 h after inoculation and used 7 days as a control.

**Fig. 2:**
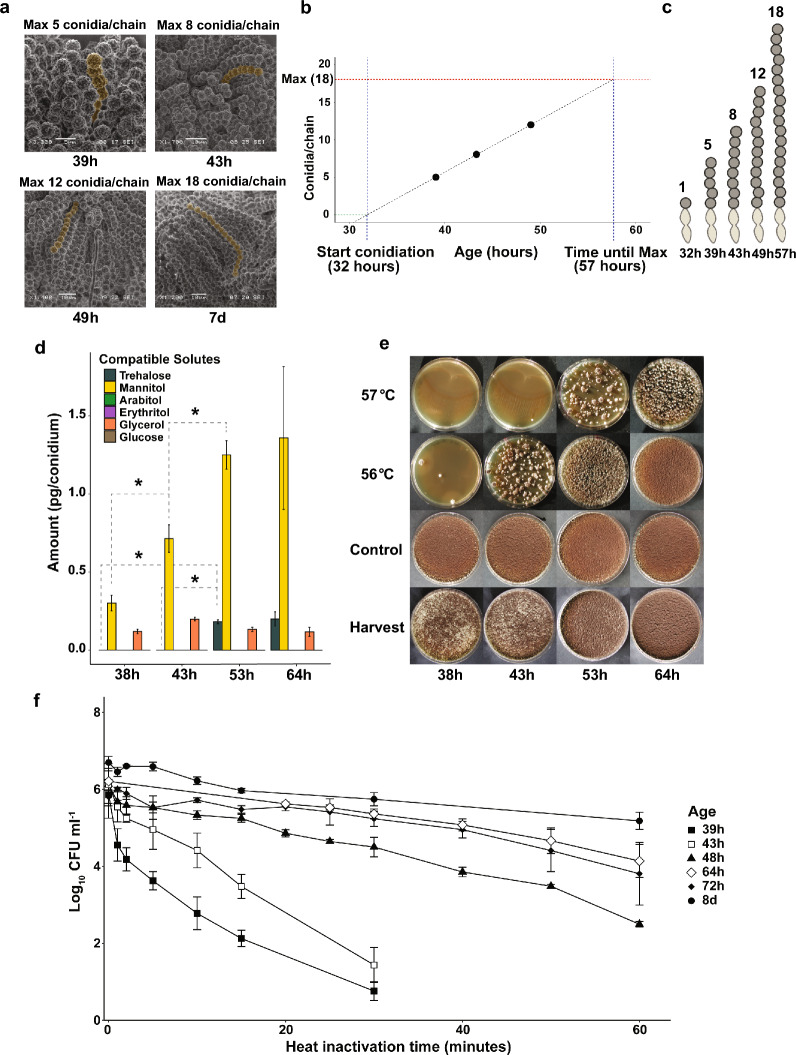
Effect of maturation on heat resistance of *Aspergillus niger* conidia. **a**, Scanning electron microscopy pictures of conidiophores of *A. niger* taken from MEA plates (in hours or days after inoculation). Spore chain lengths were manually counted for at least 15 conidiophores per sample and scanned for the longest chain. The longest possible chain per plate was noted for each time point and highlighted in the picture. **b**, The maximum chain lengths found suggests a constant conidiation speed, calculated to be around ~ 80 min/conidium. Assuming conidiation speed is constant, the first conidium was formed ~ 32 h after inoculation and the first spore chain reached its maximum length ~ 57 h after inoculation. **c**, A representation of the maximum chain lengths found for each time point. A single spore chain takes ~ 25 h to reach maximum capacity **d**, HPLC analysis of 1*10^8^ conidia revealed only a fraction of the total compatible solutes are present inside young conidia when compared to matured conidia. Trehalose and mannitol concentrations show a significant increase in time (Student’s t-test p < 0.05). **e,** Heat treatment assay results, showing that young conidia are sensitive to heat stress. 10^6^ conidia were heat treated for 10 min at either 56 °C, 57 °C or room temperature (control) and subsequently plated confluently. A picture of the harvesting plates has been included to show the level of conidiation present on the initial plates from which the conidia were harvested to perform the heat treatment assay. **f**, Inactivation curves of wild type conidia harvested at different timepoints 39 h (■), 43 h (□), 48 h (▲), 64 h (◊), 72 h (♦) and 8d (●). Heat treatments were done in a water bath at 54 °C. Samples were taken for each time point, put directly on ice and subsequently serially diluted and plated. CFUs were counted after 5 days of growth. Measurements were done in biological triplicates. Linear regression lines were drawn from these curves to calculate decimal reduction values (Table 1)

Scanning electron microscopy (SEM) pictures were taken of conidiophores from confluently plated MEA plates incubated for 39 h, 43 h, 49 h and 7 days to see how many conidia are formed during these incubation times (Fig. 2a). The largest spore chain length found from plates incubated for 7 days contained 18 conidia, in line with the 20 conidia per chain reported for *A. niger* before [33]. Based on the maximum number of conidia per chain at the different time points, spore chain growth under these conditions (MEA plates, incubated at 28 °C) was consistent at a rate of 1 conidium per 80 min (Fig. 2b, c). Extrapolation of these results suggested that the first conidium on the first phialide was formed ~ 32 h after inoculation and the spore chain reaches its maximum capacity at ~ 57 h after inoculation, indicating that the formation of a full spore chain takes around ~ 25 h under these conditions.

Additionally, the internal compatible solute concentrations and heat resistance of conidia were determined that were harvested 38 h, 43 h, 53 h and 64 h after inoculation (Fig. 2d, e). A clear correlation was found between the highly reduced compatible solutes content and heat sensitivity of young conidia. The conidia of the 38 h timepoint were considerably more heat sensitive and contained very limited amounts of compatible solutes when compared to older conidia. The heat resistance and compatible solute concentrations of the conidial population gradually increased when conidia were harvested after longer times of growth. The maximum number of conidia found on the largest spore chain at each time point was compared to the heat resistance of the conidial population. On plates where the largest spore chain contained eight conidia (43 h timepoint), conidia were still found to be heat sensitive, since no colony forming units (CFUs) were observed after exposing 10^6^ conidia for 10 min to 57 °C (Fig. 2e).

In addition to determining the sensitivity of conidia to a 10 min heat shock at 56 °C and 57 °C, heat inactivation curves were made at 54 °C to further quantify the heat resistance of differentially aged conidia (Fig. 2f). From these graphs, decimal reduction values (D_54_-values) were calculated based on the linear regression model (Table 1). The conidia from the 39 h timepoint were significantly more sensitive to heat stress (D_54_ = 5.6 ± 2.1 min) when compared to 8d conidia (D_54_ = 41.4 ± 7.3 min) and heat resistance of conidial populations gradually increased with conidial age.

**Table 1:** Heat resistance in D-values of *Aspergillus niger* conidia

Strain	Age	Temperature	D_54_ value ± SD (minutes)	D_57_ value ± SD (minutes)
wild type (N402)	8d	28 °C	41.4 ± 7.3^a^	
wild type (N402)	72 h	28 °C	36.8 ± 13.9^abcd^	
wild type (N402)	64 h	28 °C	30.8 ± 7.2^ab^	
wild type (N402)	48 h	28 °C	19.3 ± 0.3^b^	
wild type (N402)	43 h	28 °C	6.5 ± 0.5^c^	
wild type (N402)	39 h	28 °C	5.6 ± 2.1^ cd^	
wild type (N402)	8d	28 °C		6.5 ± 1.9^f^
wild type (N402)	8d	32 °C		9.7 ± 0.5^f^
wild type (N402)	8d	37 °C		17.0 ± 0.4^ g^
MA234.1	8d	28 °C	52.3 ± 25.8^abcde^	
∆*tpsABC*	8d	28 °C	22.1 ± 7.7^bce^	
∆*mpdA*, ∆*tpsABC*	8d	28 °C	3.3 ± 1.0^d^	
∆*mtdB*, ∆*tpsABC*	8d	28 °C	21.9 ± 4.2^b^	
∆*mpdA*, ∆*mtdB*, ∆*tpsABC*	8d	28 °C	22.4 ± 3.5^b^	

^a,b,c,d,e,f,g^ D-values with different superscript letters within the same column are significantly different from each other (p < 0.05). Significant differences were calculated using a Student’s T-test. D-values are listed in minutes: average ± the standard deviation. The D-values were based on a log-linear model, calculated from heat inactivation curves shown in Fig. 2f, Fig. 4c and Fig. 5c

**Fig. 3:**
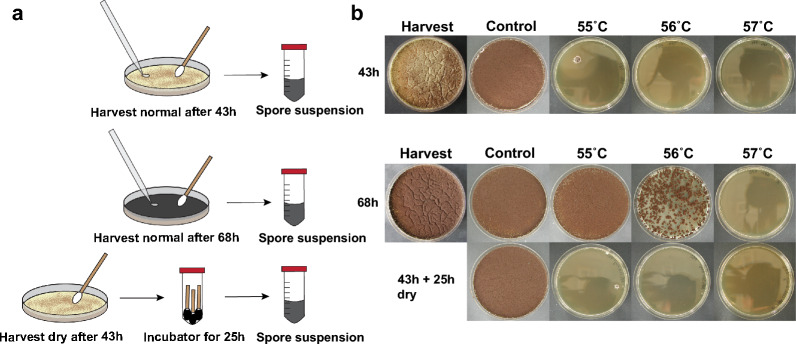
Heat resistance increase no longer observed after 43 h conidia detached from the mycelium. Three types of conidia were tested for their heat resistance: conidia from the 43 h timepoint, from the 68 h timepoint and conidia from the 43 h timepoint harvested dry and subsequently incubated for an additional 25 h. **a**, The methodology of the experiment. The 43 h and 68 h conidia were harvested in PS buffer as normal. However, some 43 h conidia were harvested dry using sterilized cotton buds and subsequently stored inside falcon tubes and returned to the 28 °C incubator for another 25 h. After 25 h, PS buffer was added, and the heat treatment assay was performed. **b**, Survival plates from the heat treatment assay. Conidia from the 43 h timepoint do not survive 10 min at 55 °C or 56 °C. In contrast, conidia from the 68 h timepoint do survive these heat treatments. When conidia from the 43 h timepoint are dry harvested and kept inside a falcon tube, returned to the incubator for another 25 h and subsequently tested for heat resistance, no increase in heat resistance is observed when compared to the conidia from the 43 h timepoint. These results show that maturation, at least in heat resistance, does no longer occur after conidia are detached, by dry harvesting, from the mycelium

We wondered if spore maturation as seen in Fig. 2 occurs when conidia were harvested at an early timepoint and kept separated from each other and from the conidiophore. To investigate this, we harvested conidia from timepoint 43 h in a way that kept them dry, and subsequently returned them to the 28 °C incubator for 25 h to compare their heat resistance with conidia from the 68 h timepoint (Fig. 3). The results showed that the maturation, in terms of heat resistance, only occurs while conidia are attached to the spore chain, since dry harvested conidia of the 43 h timepoint did not show an increase in heat resistance when incubation times were extended.

### Conidia cultivated at elevated temperatures show increased heat resistance, partly independent of trehalose content

**Fig. 4:**
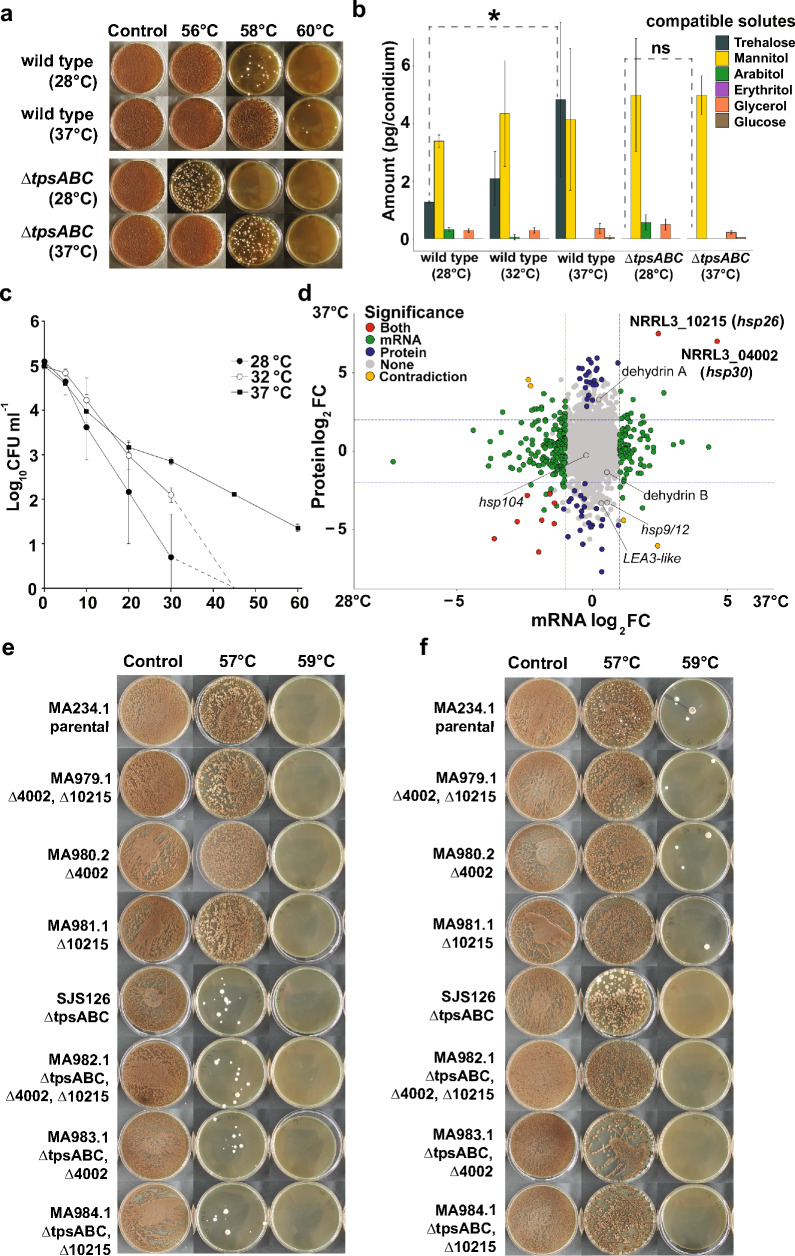
Impact of cultivation temperature on heat resistance, compatible solute profile, transcriptome and proteome of conidia. **a**, Survival plates of heat-treated conidia that were harvested from mycelium cultivated at 28 °C or 37 °C. Conidia harvested from mycelium cultivated at 37 °C are more heat resistant than conidia harvested from mycelium cultivated at 28 °C in both the wild type and the trehalose deficient strain ∆*tpsABC*. **b**, HPLC analysis showing compatible solute composition of conidia cultivated at different temperatures. Conidia of wild type show a significant (p < 0.05 tested with Student’s t-test) increase in the amount of trehalose with cultivation temperature, in contrast to trehalose deficient strain ∆*tpsABC*. **c**, Heat inactivation curves of conidia cultivated at different temperatures. Data is based on biological duplicates. D_57_-values were calculated based on linear regression lines and given in Table 1. **d**, The transcriptomes and proteomes of dormant conidia cultivated at 28 °C and 37 °C were analysed in order to find candidate genes involved in the observed heat resistance increase when cultivation temperatures are increased. Labels indicate DEGs (green), DEPs (blue) or both (red) comparing 37 °C to 28 °C cultivated conidia. Note that this graph contains only information of 2381 genes, namely genes of which proteome data was obtained, out of in total 11846 genes present on the *A. niger* NRRL3 genome. Only two genes and their corresponding proteins are significantly more present in the form of transcript and protein in 37 °C cultivated conidia versus 28 °C (NRRL3_04002 and NRRL3_10215). Knock-out strains of NRRL3_04002 and NRRL3_10215 in different backgrounds were grown for 8 days at 28 °C (**e**) or 37 °C (**f**) on MEA plates. The heat treatment assay on knock-out strains were measured in biological triplicates

Previous reports have shown that conidia cultivated at higher temperatures have increased heat resistance, and this phenotype coincides with increased trehalose concentrations [34, 35]. In order to investigate the impact of cultivation temperature on the heat resistance of conidia of *A. niger*, conidia from a wild type and a Δ*tpsABC* strain, deleted in the three trehalose-6-phosphate synthase genes, were harvested from plates that had been incubated at either 28 °C or 37 °C for 8 days and subsequently analyzed for internal compatible solute composition and heat resistance (Fig. 4a, b). Additionally, changes in heat resistance of conidia due to cultivation temperature were further quantified by following conidial heat inactivation curves (Fig. 4c). For this experiment, we increased the temperature to 57 °C to observe the potential increase in heat resistance of conidia cultivated at increased temperatures. The D_57_-values of wild type conidia cultivated at 28 °C, 32 °C and 37 °C were 6.5 ± 1.9 min, 9.7 ± 0.5 min and 17.0 ± 0.4 min, respectively (Table 1). Statistical analysis confirmed that *A. niger* conidia cultivated at 37 °C are significantly more heat stress resistant than those cultivated at 28 °C and 32 °C (p < 0.05). No significant difference was detected between D_57_-values of *A. niger* conidia cultivated at 28 °C and 32 °C (p = 0.45). This increase in heat resistance caused by increased cultivation temperature was not specific for wild type strain N402, but also observed in two other wild-type *A. niger* isolates (CBS112.32 and CBS147347) (Additional file 1: Fig. S1), showing the consistency of the increase in heat resistance due to increased cultivation temperature among *A. niger* strains. The *ΔtpsABC* strain is unable to produce trehalose and its conidia have lower heat resistance than wild type (Additional file 2: Fig. S2), but interestingly, still produced conidia with increased heat resistance upon cultivation at higher temperatures (Fig. 4a). Therefore, the observed increase in conidial heat resistance due to higher cultivation temperature is not solely due to an increase in the amount of intracellular trehalose. The other intracellular compatible solutes did not significantly alter in concentration when comparing 28 °C cultivated conidia with 37 °C cultivated conidia (Fig. 4b). We hypothesized that the observed change in heat resistance may be due to the accumulation of hitherto unidentified protective proteins. Protective proteins are abundantly present in dormant conidia [26, 30], some of which are known to impact conidial heat resistance [36].

### Transcriptome and proteome analysis of dormant conidia reveals accumulation of heat shock proteins at elevated cultivation temperature

To investigate which proteins are potentially involved in acquiring heat resistance upon cultivation at elevated temperature, transcriptome and proteome studies were conducted comparing the mRNA and protein content of dormant conidia cultivated for 8 days at 28 °C, 32 °C and 37 °C. The complete datasets containing the transcriptome and proteome results of all genes, including fold changes, are available from supplementary Additional file 9: Table S1. Principal component analysis of both the transcriptome and proteome datasets clearly indicated in both cases that the largest differences were apparent under 37 °C, while the data under 28 °C and 32 °C were comparable to each other (Additional file 3: Fig. S3). Therefore, we here discuss a comparison between the transcriptome and proteome data from conidia cultivated at 37 °C versus 28 °C, considering genes and proteins more expressed in the 37 °C condition as upregulated. In total, 1449 genes were differentially expressed when using fold change (FC) greater than 2 (FC > 2) and p-value lower than 0.05 (p < 0.05) as cut-offs. The same cut-offs were used for proteins, revealing 60 differentially expressed proteins. Of these, 666 genes and 26 proteins were upregulated, while 783 genes and 34 proteins were downregulated. Log_2_-fold changes (Log_2_FC) were calculated for both the transcriptome and proteome datasets and subsequently plotted against each other (Fig. 4d). Upregulated genes are on the top, whereas upregulated proteins are on the right. Therefore, genes that are upregulated both at the transcript level as well as on the proteome level are on the top right (Fig. 4d). Only two genes (NRRL3_04002 and NRRL3_10215) were found statistically significantly upregulated in both the transcriptome and proteome datasets. These two genes encode putative small heat shock proteins of the *hsp26/42* type [37]. All 60 differentially expressed proteins (represented by blue, pink and red dots in Fig. 4d) are listed in Additional file 10: Table S2. Additionally, an enrichment study was performed investigating the annotation terms that were over- and under-represented in the 666 upregulated genes and the 783 downregulated genes by investigating predicted PFAM domains and Gene Ontology (GO) terms. Overall, no clear biologically relevant changes could be distilled from these over- and under-represented annotation terms (see Additional file 11: Table S3).

Single deletion mutants and a double deletion mutant were constructed for the putative heat shock proteins NRRL3_04002 and NRRL3_10215 in the wild type as well as in the ∆*tpsABC* strain. The strains were cultivated at both 28 °C and 37 °C. Conidia were harvested and subsequently heat-treated and compared (Fig. 4e, f). Conidia of the knock-out strains lacking either or both putative heat shock proteins NRRL3_04002 and NRRL3_10215 showed similar heat resistance to the conidia of the parental strains. Additionally, cultivation at increased temperature (37 °C) still increased heat resistance of conidia of all knock-out strains analyzed. The use of a ∆*tpsABC* background did not alter these results. Therefore, we conclude that the two putative heat shock proteins are not important for heat stress protection of dormant conidia.

### Heat stress resistance is further reduced in conidia lacking both trehalose and mannitol

**Fig. 5:**
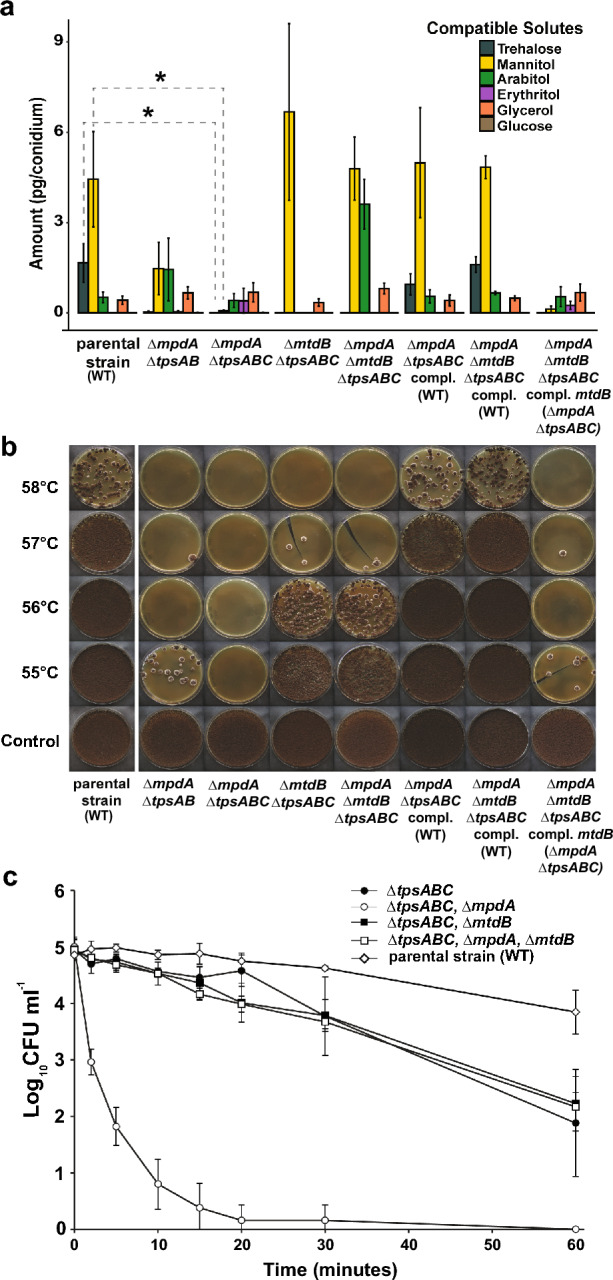
Effect of internal compatible solute composition on heat resistance of conidia. Measurements were performed in biological triplicates. **a**, Internal compatible solute composition of conidia from knock-out strains determined by HPLC analysis. Conidia from the Δ*mpdA*, Δ*tpsABC* strain contained no measurable trehalose and very little mannitol (significantly different from wild type p < 0.05 tested with Student’s t-test). The four-fold and five-fold knock-out strains were complemented back to wild type by reintroducing the genes containing two silent point mutations at the original loci. These strains show a restored compatible solute composition and conidial heat resistance comparable to wild type conidia. The five-fold knock-out strain with only the *mtdB* gene restored, re-introduces the low compatible solute composition profile. **b**, Survival plates showing CFUs of conidia from compatible solute mutants after heat treatment. Zero colonies were found testing the conidia of a Δ*mpdA*, Δ*tpsABC* strain after a relatively mild heat stress of 55 °C was applied, correlating with absent trehalose and a low mannitol concentration. **c,** Heat inactivation curves of conidia from compatible solute knock-out strains. Conidia of these strains were subjected to heat stress in a heat bath at 54 °C and sampled for up to 60 min. Mean values of three biological replicates are shown; standard deviations are indicated by error bars. D-values were calculated based on linear regression (Table 1)

To address the role of mannitol and trehalose as compatible solutes in more detail, *A. niger* strains were constructed in which the genes involved in both mannitol and trehalose biosynthesis were deleted using a CRISPR/Cas9 genome editing approach. Single knock-out strains were made in which putative mannitol dehydrogenases *(∆mtdA*, *∆mtdB*) were deleted, the mannitol-phosphate dehydrogenase *(∆mpdA*) was deleted and in which the trehalose-6-phosphate synthase encoding genes *(∆tpsA*, *∆tpsB* and *∆tpsC*) were deleted. Additional multiple knock-out strains lacking any combination of these six genes were made (Additional file 12: Table S4). Knock-out strains were also complemented by restoring the wild-type gene back to the original locus using the same CRISPR/Cas9 genome editing approach, this time targeting the knock out repair (KORE1) sequence (see Materials and Methods). Three of the complemented strains are shown in Fig. 5, namely the complementation of four-fold knock-out strain ∆*tpsABC*, ∆*mpdA*, which has the most severe phenotype in terms of conidial heat resistance and change in compatible solute profile (Fig. 5), as well as the five-fold knock-out strain ∆*tpsABC*, ∆*mpdA,* ∆*mtdB* and its complementation back to wild type and to four-fold knock-out strain ∆*tpsABC*, ∆*mpdA*. When strains were complemented back to wild type, conidia regained wild-type levels of heat resistance (Fig. 5, Additional file 3: Fig. S3). Correct deletion and complementation of all transformants was confirmed by diagnostic PCR (Additional file 4: Fig. S4).

Compatible solutes are often referred to as storage sugars, stored in high abundance within dormant spores, required for the early stages of germination [24, 38–40]. The observed reduced spore viability seen in conidia of compatible solute-deficient strains (Fig. 4) upon heat stress was likely to be due to reduced germination percentages. Thus, we investigated the germination efficiency of knock-out strains lacking compatible solutes on rich media. About 100 conidia were plated on MEA plates in triplicates to get an initial indication of conidial germination and CFUs (Additional file 13: Table S5). We observed a near 100% germination on MEA plates for wild-type conidia, as well as for all compatible solutes lacking knock-out strains reported in this study. From this result, it was concluded that all conidia lacking compatible solutes are viable and capable of germinating under the nutrient-rich conditions used in this study.

Ruijter et al*.* have shown that a Δ*mpdA* strain in *A. niger*, lacking the main mannitol biosynthesis enzyme MpdA, produces conidia containing ~ 70% less mannitol, which are more heat sensitive than wild-type [17]. These authors postulated that the residual mannitol in this strain could be produced by a reciprocal role of the mannitol dehydrogenases, which mainly catalyse the conversion from mannitol to fructose, but may also catalyse the reciprocal conversion from fructose to mannitol [41]. We confirmed that conidia from a Δ*mpdA* strain contain less mannitol (65% compared to wild type), more trehalose (300% compared to wild type) and are heat sensitive compared to conidia of the parental strain (Additional file 5: Fig. S5). Conidia of the triple knock-out strain lacking *mtdA* and *mtdB* (two putative mannitol dehydrogenases) and *mpdA* were comparable to the Δ*mpdA* strain in heat resistance and in compatible solute profile. These results indicate that indeed, only by deleting the *mpdA* gene a direct effect on the internal compatible solute composition of conidia can be observed. Surprisingly, all obtained knock-out strains lacking genes involved in the mannitol cycle were still able to produce some mannitol, indicating that additional (currently unknown) enzymes are involved in the mannitol biosynthesis of *A. niger*. The Δ*tpsA,* Δ*tpsB* and Δ*tpsC* single knock-out strains showed no significant change in compatible solute composition when compared to wild type (Additional file 2: Fig. S2). Interestingly, a small phenotype could be observed, as both conidia from a Δ*tpsA* as well as a Δ*tpsB* strain were slightly more sensitive to heat stress. Strikingly, HPLC analysis revealed that conidia of the Δ*tpsAB* and Δ*tpsABC* strains were devoid of (measurable) internal trehalose and were very heat sensitive. Therefore, both *tpsA* and *tpsB* were deemed essential for trehalose biosynthesis under these conditions.

Conidia from strains having deletions in both mannitol and trehalose biosynthesis pathways had the largest changes in compatible solute profiles (Fig. 5a). Specifically, the combination of deleting *mpdA* and the three trehalose synthase genes *tpsABC* resulted in a strain producing conidia with low concentrations of trehalose and mannitol; < 0.05 µg/conidium trehalose (instead of the wild-type 1.3 µg/conidium) and 0.05 µg/conidium mannitol (instead of the wild-type 3.4 µg/conidium). Trehalose and mannitol are normally the two most pre-dominant compatible solutes inside *A. niger* conidia. Additional deletion of the *mtdB* gene, creating the five-fold knock-out strain Δ*tpsABC*, Δ*mpdA*, Δ*mtdB*, restored mannitol concentrations to wild-type level. This unexpected result can be explained by assuming that the *mtdB* gene encodes for a mannitol dehydrogenase being able to catalyse the conversion of mannitol to fructose. As a result of deleting *mtdB,* mannitol is not readily converted to fructose, causing accumulation of mannitol in these conidia. Additionally, the five-fold knock-out strain (Δ*tpsABC*, Δ*mpdA*, Δ*mtdB*) accumulates a large amount of arabitol, indicating that if conidia are disturbed in both trehalose and mannitol biosynthesis, conidia of *A. niger* accumulate arabitol. Interestingly, this suggests that glucose-6-phosphate and fructose-6-phosphate, normally converted to trehalose and mannitol respectively, enter the pentose phosphate pathway to produce arabitol rather than entering glycolysis and subsequently synthesize glycerol (Fig. 1). All combinations in which the deletion of the *mtdA* gene was added did not alter the compatible solute composition inside conidia, and therefore strains in which *mtdA* had been additionally deleted were left out of any further experiments (Additional file 6: Fig. S6).

Heat resistance of the knock-out strains lacking multiple genes encoding proteins involved in the trehalose and mannitol biosynthesis pathways was assessed using a heat treatment assay (Fig. 5b). Using this assay, we found that four-fold knock-out strain *∆tpsABC*, *∆mpdA* produced the most heat sensitive conidia, since none of the 10^6^ conidia survived a heat treatment of 55 °C for 10 min. When compared to the compatible solute composition of these conidia (Fig. 5a), these findings confirm that near absence of both trehalose and mannitol correlate with increased heat sensitivity of conidia. Conidia of strain *∆tpsAB*, *∆mpdA*, still containing the *tpsC* gene, were more heat sensitive than wild-type conidia, but not as heat sensitive as conidia from four-fold knock-out strain *∆tpsABC*, *∆mpdA*. The presence of gene *tpsC* in this strain had a major impact on the compatible solute profile, in contrast to the previous comparison between the *∆tpsAB* and *∆tpsABC* strains (Additional file 2: Fig. S2). With *tpsC* present, the conidia from strain *∆tpsAB*, *∆mpdA* still contain mannitol, arabitol and a small but crucial amount of trehalose compared to the *∆tpsABC*, *∆mpdA* strain.

All complementation strains, having the gene deletions restored on their original locus (effectively becoming wild-type strains), restored both the compatible solute profile and the heat resistance of conidia back to wild-type concentrations (Fig. 5a, b). As an extra control, the five-fold knock-out strain Δ*tpsABC*, Δ*mpdA*, Δ*mtdB* was complemented with only the *mtdB* gene, re-creating the genotype of four-fold knock-out strain Δ*tpsABC*, Δ*mpdA*. The conidia of this complemented strain were again lacking in compatible solutes and showed the heat sensitive phenotype of the original four-fold knock-out strain (Fig. 5a, b).

Heat inactivation curves were determined, to further quantify conidial heat resistance of a subset of heat sensitive strains. Heat inactivation curves were determined using a 54 °C water bath (Fig. 5c) and D-values were calculated from linear regression lines based on this data (Table 1). The conidia of strain Δ*tpsABC,* strain Δ*tpsABC*, Δ*mtdB* and strain Δ*tpsABC*, Δ*mpdA*, Δ*mtdB*, lacking mostly trehalose but no mannitol, all displayed similar increases in sensitivity to heat compared to the parental strain with D_54_-values of 22.1 ± 7.7, 21.9 ± 4.2 and 22.4 ± 3.5 min when compared to the parental strain, which had a D_54_-value of 52.3 ± 25.8 min. In terms of spore survival, these three knock-out strains display a 3-log reduction after 60 min (1 in 10^3^ conidia survives the heat treatment) when compared to 1-log reduction (1 in 10^1^ conidia survive the heat treatment) observed in the parental strain. The conidia of the four-fold knock-out strain *∆tpsABC*, *∆mpdA* were the most heat sensitive, with a decimal reduction value of D_54_ = 3.3 ± 1.0 min when compared to the parental strain D_54_ = 52.3 ± 25.8 min. Therefore, a 52 min heating at 54 °C results in a ~ 1 log reduction in the amount of viable conidia of the parental strain (1 in 10^1^ conidia survives the heat stress), whereas this heat treatment would result in an almost 16 log reduction in the amount of viable conidia of the *∆tpsABC*, *∆mpdA* strain (1 in 10^16^ conidia survives the heat stress). These results in combination with the obtained compatible solute profiles (Fig. 4a) suggest that both mannitol and trehalose play crucial roles in the heat resistance of *A. niger* conidia.

## Discussion

We show, using model organism *A. niger*, that chains of conidia gradually increase in length, and that the heat resistance of the spore population increases gradually in time. Unmatured conidia contain low concentrations of trehalose and mannitol, rendering them heat sensitive compared to their older counterparts (Fig. 2). The heat resistance of conidia gradually increases during spore maturation, correlating with the accumulation of compatible solutes. Here, we show for the first time that compatible solutes accumulate during the spore maturation process.

Spore maturation in terms of heat resistance requires attachment to the spore chain, since pre-emptive detachment from the spore chain results in heat sensitive conidia that no longer increase their heat resistance (Fig. 3). It is yet unclear why attachment to the spore chain is important for spore maturation. However, this finding ties in nicely with a recent study performed in *A. nidulans* which showed that conidia are still transcriptionally active after their formation, if still attached to the spore chain [31]. These findings suggests that de-attachment of conidia from the spore chain could be the trigger for entry into dormancy, either by de-attachment itself or the spore’s dehydration during this process [42].

Our findings suggest that compatible solute compositions adapt to environmental factors in a certain time window, namely after spore formation but before detachment. Conidia harvested from plates cultivated at a high(er) temperature have high(er) heat resistance in both *A. fumigatus* and *P. roqueforti*, also seen here for *A. niger*, and this heat resistance increase correlates with an increase in the amount of internal trehalose in these three species [34, 35]. Our study shows that the increased accumulation of trehalose inside conidia due to high cultivation temperature occurs after conidial formation when the conidia are still attached to the spore chain. It is currently not known how conidia accumulate compatible solutes after formation, since limited glucose is measured in unmatured conidia of the 38 h timepoint. Although no major GO-terms were over- or underrepresented in the transcriptome and proteome datasets, both a 1,3-betaglucosidase and N-acetyl-glucosamine-6-phosphate deacetylase are upregulated in the proteome comparison between 37 °C and 28 °C cultivated conidia (Additional file 10: Table S2). We speculate that, during maturation, conidia can re-route their resources by sacrificing building blocks meant for cell wall biosynthesis, such as N-acetyl glucosamine, to function as a sugar resource for compatible solute accumulation. The spore’s cell wall are still subject to change after spore formation, and also show a gradual change during spore maturation on the spore chain [43]. Additionally, previous research has shown that increased cultivation temperature causes reduced melanin concentrations in the cell wall of *A. fumigatus* conidia [35]. However, more research is needed to determine the energy source used for additional compatible solute accumulation during maturation of conidia when subjected to increased temperatures.

To identify possible candidate proteins involved in the acquired heat resistance by cultivation at higher temperatures, we performed both transcriptome and proteome studies on dormant conidia of *A. niger* cultivated at 28 °C, 32 °C or 37 °C. Only two genes show upregulation in both transcriptome and proteome datasets, NRRL3_04002 and NRRL3_10215, which both code for putative small heat shock proteins. A recent paper describes how the (best hit) homologues of these two small heat shock proteins in *A. nidulans*, named *hsp26* (AN7892) and *hsp30* (AN2530), are significantly upregulated when conidia attached to the spore chain are heat treated for 4 h [31]. This again emphasizes the potential role of these two small heat shock proteins in the heat resistance of conidia as a response to high temperatures. However, the *Δ04002* and Δ*10215* strains, made in either a wild-type or Δ*tpsABC* background, did not show altered heat resistance phenotypes compared to their parental strains. These findings suggest that the increased heat shock protein production might not aid in the heat resistance of dormant conidia and is not responsible for the increase in heat resistance of conidia seen when cultivation temperatures are increased. It is possible that the heat resistance of the subsequent germlings and mycelium is altered by the lack of these small heat shock proteins. Interestingly, a recent study investigating the breaking of dormancy of *S. cerevisiae* ascospores revealed a novel function of small heat shock protein Hsp42, acting as a chaperone to ensure proper re-solubilization of the spore’s proteome during germination [44]. Therefore, the accumulation of small heat shock proteins as observed in this study could be needed for proper re-solubilization of the proteome during germination, although more research is needed to investigate this point.

In our study, putative protective proteins previously suggested to be linked to conidial stress resistance were deleted in order to investigate their role in conidial heat resistance (Additional file 7: Fig. S7). This includes homologues of two dehydrin-like proteins DprA and DprB [45], a homologue of ConJ [46], a homologue of Hsp9/12 and another LEA3-like protein [26, 47, 48] as well as the Hsp104 homologue [36, 49, 50]. Of these, only the ∆*hsp104* strain produced conidia with lowered heat resistance, which is in line with recent observations made in *Fusarium pseudograminearum* [36]. Importantly, none impacted the heat resistance increase observed during cultivation at elevated temperatures (Additional file 7: Fig. S7). The heat resistance of conidia from the ∆*hsp104* strain is comparable to the heat resistance of the ∆*tpsABC* strain and is therefore still significantly higher than the heat resistance of the ∆*tpsABC*, ∆*mpdA* strain (Fig. 5C). Deletions of these protective proteins did not alter the conidial compatible solute profiles (Additional file 7: Fig. S7c).

Deletion of the genes *mpdA*, *mtdA* and *mtdB* resulted in conidia that were still able accumulate mannitol (conidia of triple knock-out strain contained ~ 80% of wild-type mannitol concentration) (Additional file 5: Fig. S5). Previously, it was suggested that the mannitol dehydrogenases can act to either synthesize or metabolize mannitol, which would explain the remaining ~ 30% mannitol observed in the Δ*mpdA* strain when compared to wild type [17]. However, the conidia of a strain in which the two putative mannitol dehydrogenases (*mtdA*, *mtdB*) have been additionally deleted still contain mannitol, suggesting that either additional mannitol dehydrogenases are present, or an alternative biosynthesis route is producing the leftover mannitol in these strains. The *mtdA* deletion did not alter mannitol concentrations whereas the *mtdB* deletion did (Additional file 6: Fig. S6), suggesting that *mtdB* rather than *mtdA* codes for the main mannitol dehydrogenase in *A. niger*. Therefore, a mannitol cycle could still be active in *A. niger,* contrasting a previous report stating that such cycle does not exist based on the spatial differentiation of the MpdA and MtdA enzymes [25].

The roles of *tpsC* and *mtdB* determining the compatible solute composition of conidia only became apparent when multiple enzymes in both the trehalose and mannitol biosynthesis pathway were disrupted. Initially, data of strains only deleted in genes part of the trehalose biosynthesis pathway (*tpsABC*), suggested that *tpsC* is not important for trehalose biosynthesis (Additional file 2: Fig. S2). However, the presence of the *tpsC* gene did have a significant impact on the internal compatible solute composition and heat resistance when a Δ*tpsAB,* Δ*mpdA* strain was compared with a Δ*tpsABC,* Δ*mpdA* strain (Fig. 5). Therefore, only when genes were deleted in both the trehalose and mannitol biosynthesis pathways was the importance of *tpsC* for the biosynthesis of trehalose observed. Similarly, the importance of *mtdB* for the metabolism of mannitol was only observed in a strain disrupted in both the trehalose and mannitol biosynthesis pathways (Additional file 6: Fig. S6). These observations clearly indicate that it is important to take presence and absence of paralogous genes into consideration while analyzing their respective functions in related fungal strains. E.g. in *Aspergillus fumigatus*, it was found that *tpsA-B* were important for trehalose biosynthesis and *tpsC-D* were not. Similarly in *Aspergillus nidulans* only *tpsA* was found important for trehalose biosynthesis under the tested conditions [18, 51].

The D_54_-value of 39 h conidia, lacking most compatible solutes, and the D_54_-value of strain Δ*mpdA*, Δ*tpsABC*, lacking all trehalose and most mannitol, were 5.6 ± 2.1 min and 3.3 ± 1.0 min, respectively. The conidia of the 38 h timepoint are not identical to conidia of an 8 days old timepoint from a Δ*tpsABC*, Δ*mpdA* strain in terms of compatible solute composition and heat resistance, but do show similar trends. Both contain low concentrations of compatible solutes and are very heat sensitive with comparable D-values. These D_54_-values are significantly lower than those observed in 18 natural strains of *A. niger* that ranged from 9.4 ± 0.85 to 50.4 ± 11.9 min [52].

## Conclusions

Compatible solutes accumulate during conidia maturation process. This finding suggests that compatible solute profiles are established during this time window, after formation of the first conidium. *A. niger *conidia lacking most compatible solutes, either because they are young or because they are lacking the genes necessary for trehalose and mannitol biosynthesis, are very heat sensitive (16 orders of magnitude). Transcriptome and proteome analyses on conidia cultivated at elevated temperatures revealed accumulation of two small heat shock proteins. However, simultaneous deletion of the genes encoding these heat shock proteins did not exhibit altered heat resistance. Taken together, we conclude that the compatible solutes trehalose and mannitol are the major determinants of conidial heat resistance.

## Materials and methods

### Growth conditions and media

The strains used in this study are listed in Additional file 12: Table S4. Media were prepared as described previously [53]. Minimal medium (MM) contained 1% (w/v) glucose and 1.5% agar, supplemented with hygromycin (100 µg ml^−1^) when needed. Transformation plates consisted of minimal medium + sucrose (MMS), contained 32.5% (w/v) sucrose and 1.5% agar, supplemented with hygromycin (200 µg ml^−1^) and caffeine (500 µg ml^−1^). Malt extract agar (MEA, Oxoid) contained 3% (w/v) malt extract, 0.5% (w/v) mycological peptone and 1.5% agar. To harvest conidia, strains were first inoculated on MEA plates and grown for 8 days at 28 °C, unless noted otherwise. Conidia were harvested by adding 13 ml of physiological salt buffer (PS, 0.9% (w/v) NaCl and 0.02% (v/v) Tween-80 in demi water), after which the conidia were carefully scraped from the plate using a cotton swab. The resulting spore solution was filtrated through a sterilized filter (Amplitude™ Ecocloth™ Wipes, Contec Inc., Spartanburg, SC, USA) to remove mycelial debris.

### Conidial age experiments

The age of conidia was defined as the amount of time passed since inoculation. Hence, conidia harvested from a plate that had been incubated for 38 h are referred to as 38 h conidia. The precise amount of time passed may vary a maximum of 15 min from their designated age. To prevent heterogeneity in the spore population, conidia were plated out confluently. All plates were inoculated confluently using sterilized glass beads and subsequently incubated at 28 °C. All conidial age experiments were performed using MEA plates.

### CRISPR/Cas9-mediated genome editing approach

A previously described marker-free CRISPR/Cas9 mediated gene editing protocol was used to make genetic alterations [54]. Deletions were made in the non-homologous end-joining (NHEJ)-deficient *A. niger* background, strain MA234.1 [55]. Genomic DNA was extracted from purified transformants using a phenol–chloroform based protocol [53]. Diagnostic PCR amplifying the deleted region was performed to check for correct deletion of the target gene. All primers used in this study are listed in Additional file 14: Table S6. The sgRNAs targeting the gene of interest (GOI) were created using CHOPCHOP predictors [56]. All plasmids used in this study are listed in Additional file 15: Table S7. The DNA repair fragments were obtained by amplifying both the 5’ and the 3’ flanks of the GOI from the parental strain MA234.1, followed by fusion PCR. Primers contained an overhang, creating a novel 23 base pairs region replacing the original open reading frame (ORF). This 23 base pairs overhang was identical for all deletions and resulted in the replacement of the original gene by a new unique artificially created CRISPR/Cas9 target sequence named KORE1 (sequence: CCGGCTTATATTGGTACCACTCC). Complementation of the knock-out strains was done by re-inserting the original gene on the original locus, using a CRISPR/Cas9 containing vector targeting the KORE1 sequence to cut open the original loci. In this case, repair DNA was a PCR product amplified from the original gene including both 5’ and 3’ flanks with the following alteration: In order to show that the correct complementation was obtained, two silent mutations were introduced into the original gene (created during amplification by using primers with overhangs containing these point mutations) before re-inserting the original gene into the genome. In this way, sequencing of the gene confirms that the obtained strain is indeed complemented, and not a contamination of the genetically identical original parental strain MA234.1.

### HPLC analysis

HPLC analysis was performed following a previously established protocol [13]. In short, 10^8^ conidia inside a 2 ml Eppendorf safe-lock tube were centrifuged and supernatant was removed. The pellets were flash frozen in liquid nitrogen, and two stainless steel beads (diameter 3.2 mm) were added per tube. The tubes were loaded into a TissueLyser II adapter (pre-cooled in -80 °C) and conidia were cracked using a TissueLyser II (QIAGEN) shaking at 30 Hz for 6 min. After cracking 1 ml Milli-Q water was added and samples were heated in a 95 °C water bath for 30 min. Samples were centrifuged for 30 min and the supernatant was filtered (0.2 µm Acrodisc). Samples were stored at -20 °C until HPLC analysis.

HPLC analysis was done using two Sugar-Pak I columns (Waters), placed in line to get good separation between polyols. A sample volume of 20 µl was injected in the mobile phase consisting of 0.1 mM Ca EDTA in ultrapure water and samples were followed for 30 min. A mixture of trehalose, glucose, glycerol, erythritol, mannitol and arabitol (0.002% – 0.10% w/v) was used as reference. All calibration curves showed an R2 > 0.999 with a limit of detection.

### RNA isolation and initial steps of protein isolation

To obtain dormant conidia, MEA plates were inoculated confluently using sterilized glass beads and incubated at 28 °C, 32 °C and 37 °C for 8 days. Temperatures of the incubators were double-checked with multiple thermometers and settings were adjusted if needed to obtain cultivation temperatures as close as possible to absolute temperatures. Conidia were harvested in cold PS + 0.02% Tween 80 buffer and filtered using sterile filters (Amplitude Ecocloth, CONTEC) into a falcon tube and put directly on ice. In the case of plates cultivated at 37 °C, conidia from three plates were pooled (forming 1 pellet) in order to get enough conidia per sample. Samples were centrifuged for 5 min at 3000 rpm and 4 °C, after which supernatant was removed from the pellets. The pellets were resuspended in 100 µl RNAlater (Sigma) and subsequently flash frozen in liquid nitrogen. Stainless steel grinding jars for Tissuelyzer use (QIAGEN) were pre-cooled in a − 80 °C freezer and kept cold with liquid nitrogen inside a mortar until the flash frozen pellets were added. Cells were broken using a Tissuelyzer II (QIAGEN) shaking for 1 min at 30 Hz. Crushed samples, while still cold and powdery, were divided between two safe-lock Eppendorf tubes; one meant for proteome analysis (kept in liquid nitrogen and subsequent − 80 °C until further analysis) and one for RNA extraction already containing 450 µl RLC buffer from the RNeasy Plant Mini Kit (QIAGEN). RNA extraction and purification was continued following the manual supplied by the manufacturer including the on-column DNA digestion step. RNA quality was assessed on gel and the quantity was determined using Qubit (ThermoFisher). The RNA samples were handed over to the Utrecht Sequencing facility for Illumina NextSeq2000 2 × 50 paired-end sequencing.

### Protein sample preparation

Protein extraction was performed on powdered samples using an extraction buffer of 1% (w/v) sodium dodecyl sulfate (SDS, Sigma-Aldrich) in 100 mM ammonium bicarbonate (AMBIC, Sigma-Aldrich), by vigorous vortexing and sonication followed by 10 min of centrifugation at 10,000 g to clear the extract. Supernatants were transferred to a fresh tube and their protein content was determined by bicinchoninic acid assay (BCA assay, Thermo Scientific, Etten-Leur, the Netherlands), according to the manufacturer’s protocol.

Subsequently, 20 μg of protein for each sample was reduced and alkylated by the addition of 10 mM Tris carboxyethyl phosphine (TCEP, Sigma-Aldrich) and 40 mM chloroacetamide (CAA, Sigma Aldrich) and incubation at 60 °C for 30 min. Samples were cooled at room temperature and cleaned up using protein aggregation capture on microparticles to remove interfering contaminants such as SDS by protein precipitation [57] on carboxyl modified magnetic beads (1:1 mixture Sera-Mag A and Sera-Mag B Thermo Scientific, Etten-Leur), also called SP3 [58] using improvements described previously [59].

In short, 20 μg of protein lysate (20 μl) was added to 2 μl (100 μg/μl beads suspension) of carboxyl modified magnetic bead mixture. The mixture was brought to 50% (v/v) acetonitrile (ULC-MS grade, Biosolve) concentration, vortex mixed and incubated for 20 min at room temperature, after which samples were placed on a magnetic rack. Supernatants were removed and beads were washed twice with 200 μl 70% (v/v) ethanol (HPLC-Grade, Biosolve) on the magnetic rack. Subsequently, beads were washed with 180 μl of acetonitrile on the magnetic rack and following removal, air dried. Beads were resuspended in 20 μl of digestion buffer (100 mM AMBIC), and following addition of 1 μg of trypsin (sequencing grade, Promega, 1:20 enzyme to substrate ratio by weight) and incubation overnight at 37° C. Following digestion, supernatants were acidified by addition of 1% (v/v) formic acid (ULC-MS Biosolve) and careful transfer of peptide containing supernatants to a clean tube after placing samples on a magnetic rack. Cleaned samples were ready for LC–MS analysis.

### Mass spectrometry (proteome)

200 ng of peptides were injected onto a 75 μm × 250 mm column (C18, 1.6 μm particle size, Aurora, IonOpticks, Australia) kept at 50 ℃ at 400 nl/min for 15 min with 3% acetonitrile, 0.1% formic acid in water using an Ultimate 3000 RSLCnano UHPLC system (Thermo Scientific, Germering, Germany). Peptides were subsequently separated by a multi-step gradient to 40% acetonitrile at 90 min, 99% acetonitrile at 92 min held until 102 min returned to initial conditions at 105 min and kept there until 120 min to re-equilibrate the column. Eluting peptides were sprayed into a captive spray source (Bruker, Bremen, Germany) with a capillary voltage of 1.5 kV, a source gas flow of 3 l/min of pure nitrogen and a dry temperature setting of 180 °C, attached to a timsTOF pro (Bruker, Bremen, Germany) trapped ion mobility, quadrupole, time of flight mass spectrometer. The timsTOF was operated in PASEF mode of acquisition. The TOF scan range was 100–1700 m/z with a tims range of 0.6–1.6 V·s/cm2. In PASEF mode a filter was applied to the m/z and ion mobility plane to select features most likely representing peptide precursors, the quad isolation width was 2 Th at 700 m/z and 3 Th at 800 m/z, and the collision energy was ramped from 20–59 eV over the tims scan range to generate fragmentation spectra. A total number of 10 PASEF MS/MS scans were used, scheduled with a total cycle time of 1.16 s, a scheduling target intensity of 2 × 10^4^ and intensity threshold of 2.5 × 10^3^ and a charge state range of 0–5. Active exclusion was on (release after 0.4 min), reconsidering precursors if ratio current/previous intensity > 4.

### Data analysis (proteome)

Generated mass spectral data were processed using MaxQuant (Version 1.6.10.43), searching a proteome database of *Aspergillus niger* (Uniprot downloaded September 2019). The proteolytic enzyme was set to trypsin allowing for a maximum of two missed cleavages. Carbamidomethyl (C) was set as a fixed modification Oxidation (M) as a variable modification. Group specific settings timsDDA, and LFQ and iBAQ for quantitation were enabled. Matched between runs was also enabled using the standard settings for matching. Fold changes were calculated by dividing the average LFQ intensities of conidia cultivated at 37 °C by the average LFQ intensities of conidia cultivated at 28 °C. Significantly differentially expressed proteins (DEPs) were calculated using the DEP package in R [60] using the user defined cut-offs alpha = 0.05 and lfc = log_2_(2).

### Data analysis (transcriptome)

Raw transcriptome data was obtained from the Utrecht Sequencing facility. The fastq files were mapped to the publicly available *A. niger* NRRL3 genome [61] using HISAT2 [62] with intron lengths set between 20 and 1000 and suppressing SAM records for reads that failed to align. SAM files were turned into BAM files using the view and sort functions of SAMtools [63]. The htseq-count option of the tool HTSEQ [64] was used to create count text files. The count files served as input for the DESeq2 R package [65] using the DEseqDataSetFromHTSeqCount option. Shrunken log fold changes were calculated using the apeglm package [66].

The log_2_ fold changes (log_2_FC) in transcriptome and proteome data were compared using only 2381 genes (out of the 11863 genes present on the *A. niger* NRRL3 genome), of which both transcriptome and proteome data was available. Enrichment studies were done on the transcriptome dataset. Custom scripts were developed in Python and implemented in a web interface (https://fungalgenomics.science.uu.nl) to analyze over- and under-representation of functional annotation terms in sets of differentially regulated genes using the Fisher Exact test. The Benjamin-Hochberg correction was used to correct for multiple testing using a p-value < 0.05.

### Heat treatment assays using a thermo-cycler

Heat treatment assays were applied to test for changes in the heat resistance of conidia, see Additional file 8: Fig. S8 for a detailed overview. Conidia were harvested from confluently plated MEA plates grown for 8 days at 28 °C unless noted otherwise. Measurements were done in biological triplicates. Freshly harvested conidia were diluted in PS buffer and spore suspension concentrations were measured using a Bio-Rad TC20™ automated cell counter. A total of 1*10^6^ conidia inside a volume of 100 µl PS buffer was used for each heat treatment assay. The heat treatment was applied in a thermocycler for 10 min at 55 °C, 56 °C, 57 °C or 58 °C. Controls were subjected to room temperature for 10 min. After heat treatment, the conidia were confluently plated on MEA containing 0.05% (v/v) Triton^®^ X-100 and incubated for five days at 28 °C, after which pictures were taken. The number of colony forming units (CFUs) per plate is considered a readout of the amount of conidia that survived the heat treatment.

### Heat inactivation experiments using a water bath

To further quantify the heat resistance, inactivation curves of selected knock-out strains were made. These measurements were done in biological triplicates. Freshly harvested conidia were diluted in PS and spore concentrations were measured using a Bio-Rad TC20™ automated cell counter. A volume of 19.8 ml of PS buffer inside an Erlenmeyer was pre-heated in a water bath (Julabo Corio c-bt27) to 54 °C. A total volume of 200 µl spore suspension was added to obtain a final concentration of 1 × 10^7^ conidia ml^−1^ inside each Erlenmeyer for the heat inactivation experiment. Samples were taken after 0, 2, 5, 10, 15, 20, 30 and 60 min of exposure to heat stress. The samples were serially diluted into 10^6^, 10^5^, 10^4^ and 10^3^ conidia ml^−1^, after which 100 µl of each dilution was inoculated on MEA containing 0.05% (v/v) Triton^®^ X-100. Plates were incubated for seven days at 30 °C, after which the number of colony forming units (CFUs) per plate was counted. The results were used to calculate and subsequently plot inactivation curves based on a log-linear fit. The linear regression line in turn was used to calculate a decimal reduction value (D-value).

### Supplementary Information

**Additional file 1: Figure S1 **Cultivation temperature impacts heat resistance of wild-type A. niger strains CBS112.32 and CBS147347. Heat resistance was investigated using the heat treatment assay, in which conidia are harvested, diluted and subsequently heat treated for 10 minutes in a thermocycler after which 106 conidia are plated. Pictures were taken after 5 days of incubation at 28 °C. a, Heat resistance of wild-type strain A. niger CBS112.32. Conidial heat resistance increases when conidia were harvested from plates cultivated at 37 °C versus 28°C as seen by the colony forming units obtained after 10 minutes at 58 °C. b, Heat resistance of wild-type strain A. niger CBS147347. Conidial heat resistance increases when conidia were harvested from plates cultivated at 37 °C versus 28 °C as seen by the colony forming units obtained after 10 minutes at 60 °C.

**Additional file 2: Fig. S2 **Internal compatible solute composition and heat resistance of conidia from A. niger trehalose knock-out strains. Conidia were freshly harvested from MEA plates grown for 8 days at 28°C. Measurements were performed in biological triplicates. a, Internal compatible solute composition of conidia from trehalose knock-out strains as determined by HPLC analysis. Conidia from ΔtpsAB and ΔtpsABC strains were significantly impacted in their internal compatible solute composition, as they produced no measurable amount of trehalose. No significant increase in any other type of sugar was measured in these two strains. b, Heat treatments were applied for 10 minutes to 106 conidia. After heat treatments conidia were plated on plates containing MEA+0.05% triton x-100. Plates were grown for 5 days at 28°C after which the pictures were made as shown above. All conidia from knock-out strains lacking tps genes showed at least a slight decrease in heat resistance. However, the largest drop in heat resistance was observed in strains ΔtpsAB and ΔtpsABC. This drop in conidial heat resistance corresponds with the decrease and overall absence of trehalose inside these conidia. Overall, a clear link is seen between internal trehalose concentration and conidial heat resistance.

**Additional file 3: Figure S3.** Heat shock results of strains complemented to have a wild type genotype. Conidia were freshly harvested from MEA plates grown for 8 days at 28°C. Measurements were performed in biological triplicates. Complemented strains were checked for two silent point mutations introduced into the genes (see Materials and Methods) to distinguish between the original wild type and the complemented strains. All knock-out strains were successfully complemented back to wild type genotype and their conidia show wild type levels of heat resistance.

**Additional file 4: Figure S4 **Principal component analysis (PCA) on the transcriptome and proteome datasets. The three cultivation conditions; 28 °C, 32 °C and 37 °C were compared. Most of the variance can be explained by the x-axis, of which the 37 °C condition is the largest contributing factor in both cases. a, PCA of the transcriptome data, 84% of the variance is found on the x-axis which is mostly due to the 37°C condition. b, PCA of the proteome data. Variance is not as large as in the transcriptome dataset, but most of it is due to the x-axis difference which is due to the 37°C condition.

**Additional file 5: Fig. S5 **Diagnostic PCR confirming deletions in knock-out strains. Each column on the gel represents an amplification product from the wild type strain MA234.1, a gene knock-out strain, or the GeneRuler ladder. a, Deletion of the tpsA gene. Amplification was done using diagnostic primers DIAG_tpsA_5'_fw and DIAG_tpsA_3'_rv. If the gene is present, a band size of 4118 bps is expected. If the gene is absent, a band size of 2189 bps is expected. b, Deletion of the tpsB gene. Amplification was done using diagnostic primers DIAG_tpsB_5'_fw and DIAG_tpsB_3'_rv. If the gene is present, a band size of 3994 bps is expected. If the gene is absent, a band size of 2281 bps is expected. c, Deletion of the tpsC gene. Amplification was done using diagnostic primers DIAG_tpsC_5'_fw and DIAG_tpsC_3'_rv. If the gene is present, a band size of 3894 bps is expected. If the gene is absent, a band size of 2196 bps is expected. d, Deletion of the mpdA gene. Amplification was done using diagnostic primers DIAG_mpdA_5'_fw and DIAG_mpdA_3'_rv. If the gene is present, a band size of 2499 bps is expected. If the gene is absent, a band size of 1345 bps is expected. e, Deletion of the mtdA gene. Amplification was done using diagnostic primers DIAG_mtdA_5'_fw and DIAG_mtdA_3'_rv. If the gene is present, a band size of 2176 bps is expected. If the gene is absent, a band size of 1387 bps is expected. f, Deletion of the mtdB gene. Amplification was done using diagnostic primers DIAG_MtdB_5'_fw and DIAG_MtdB_3'_rv. If the gene is present, a band size of 4233 bps is expected. If the gene is absent, a band size of 2347 bps is expected.

**Additional file 6: Fig. S6 **Internal compatible solute composition and heat resistance of conidia from A. niger mannitol cycle knock-out strains. Conidia were freshly harvested from MEA plates grown for 8 days at 28°C. Measurements were performed in biological triplicates. a, Internal compatible solute composition of conidia from mannitol knock-out strains lacking genes involved in the mannitol cycle. Only the deletion of the mpdA gene resulted in a change of internal sugar composition inside conidia of A. niger. Strain ΔmpdA contains less mannitol and more trehalose as has been observed before (17). The internal compatible solute composition of the strain ΔmpdA, ΔmtdAB was the same as observed for ΔmpdA. As such, no effect of both mtdA or mtdB deletion on compatible solute composition of conidia was seen in these knock-out strains. b, Heat treatments were applied for 10 minutes to 106 conidia. After heat treatment, conidia were plated on plates containing MEA+0.05% triton x-100. Plates were grown for 5 days after which the pictures were made as shown above. The mpdA deletion has the largest effect on the heat resistance of A. niger conidia, as the number of observed CFUs is less than 100 when heat stress of 57°C is applied. The strain in which mpdA and both mannitol dehydrogenases mtdA and mtdB have been knocked out, is comparable in heat resistance to the ΔmpdA single knock-out strain, suggesting that mtdA and mtdB do not significantly contribute to the heat resistance of A. niger conidia.

**Additional file 7: Fig. S7 **The mtdA gene does not have an impact on internal compatible solute composition of conidia. The genes mtdA and mtdB have been deleted in many different backgrounds. Here we give an overview of the effect of the individual mutations on the internal compatible solute profiles of conidia. In all cases, the parental strain without the mtdA or mtdB mutation is given first, followed by the strain in which the mtdA or mtdB gene is additionally knocked-out. a, Here the effect of the mtdA deletion is visualized. When comparing ΔmtdA strains with their respective parental strains, no significant changes in compatible solute profiles were found in any of the knock-out strains where an extra ΔmtdA deletion was made. b, Here the effect of the mtdB deletion is visualized. Initially, in single double or triple knock-out strains the absence of the mtdB gene does not show any significant impact on the compatible solute profiles. However, when deleting mtdB in the ΔmpdA, ΔtpsABC strain, therefore becoming strain ΔmpdA, ΔmtdB, ΔtpsABC, a clear change in compatible solute profile is observed.

**Additional file 8: Fig. S8 **Heat treatment assay protocol. All strains were plated homogenously and subsequently grown on MEA for 8 days at 28°C, unless noted otherwise. Conidia were harvested in PS buffer and subsequently counted using a Bio-Rad Automated Cell Counter. A total volume of 100 µl containing 1*106 conidia were heat treated per PCR tube in a thermocycler for 10 minutes. After heat treatment the 100 µl is plated homogenously on MEA + 0.05% Triton X-100 and grown for 5 days at 28°C after which CFUs are counted and pictures were taken.

**Additional file 9: Table S1**. Transcriptome and proteome data.

**Additional file 10: Table S2. **The 60 proteins significantly more or less present in conidia cultivated at 37°C. Positive values (green) in Log2 fold changes found significant (p < 0.05) show upregulation in conidia cultivated at 37°C, whereas negative values (red) show downregulation in conidia cultivated at 37°C. Descriptions are based on EuKaryotic Orthologous Groups (KOG) found as part of MycoCosm on the JGI website 68. All descriptions are putative and solely based on homology. The baseMean DESeq2 values represent the average of normalized counts and the LFQ intensities represent quantified proteome data based on peptides found (higher values = more protein present). Normalization, Log2FC and their significance were calculated with the DESeq2 package in R for the transcriptome data and the DEP package in R for the proteome data.

**Additional file 11: Table S3. **Enrichment analysis. Here, we compared the set of significantly up- and down-regulated genes when comparing conidia cultivated at 37°C versus conidia cultivated at 28°C. no clear biologically relevant changes could be distilled from these over- and under-represented annotation terms.

**Additional file 12: Table S4. **List of strains used in this study.

**Additional file 13: Table S5. **Colony forming units of A. niger conidia from knock-out strains plated on MEA.

**Additional file 14: Table S6**. List of primers used in this study.

**Additional file 15: Table S7**. Plasmids used in this study.

## Data Availability

All data is included in the article and/or supplementary material. Strains, plasmids and primers are available upon request. The RNA-seq expression data is available from the NCBI SRA database under BioProject ID PRJNA981668. Mass spectrometry data for proteomics have been deposited at ProteomeXchange (PXD042851) and the Massive Repository (MSV000092133).
