# Supplementary figures and images for: Compatible solutes determine the heat resistance of conidia
Source: Fungal Biol Biotechnol. 2023 Nov 13;10:21. doi: 10.1186/s40694-023-00168-9 (PMC10644514; doi:10.1186/s40694-023-00168-9)

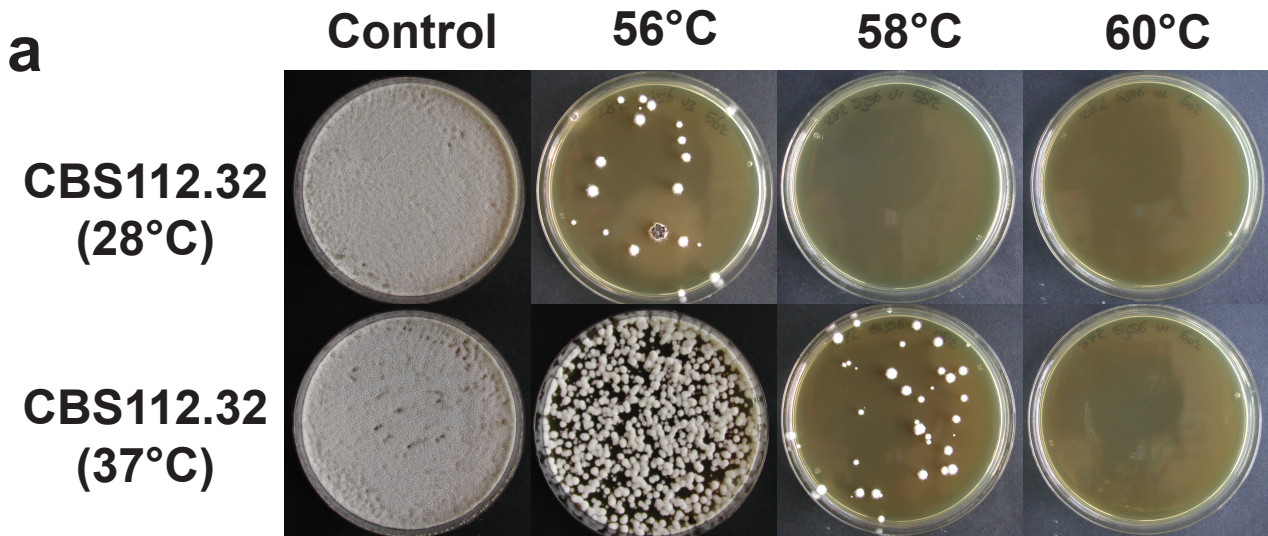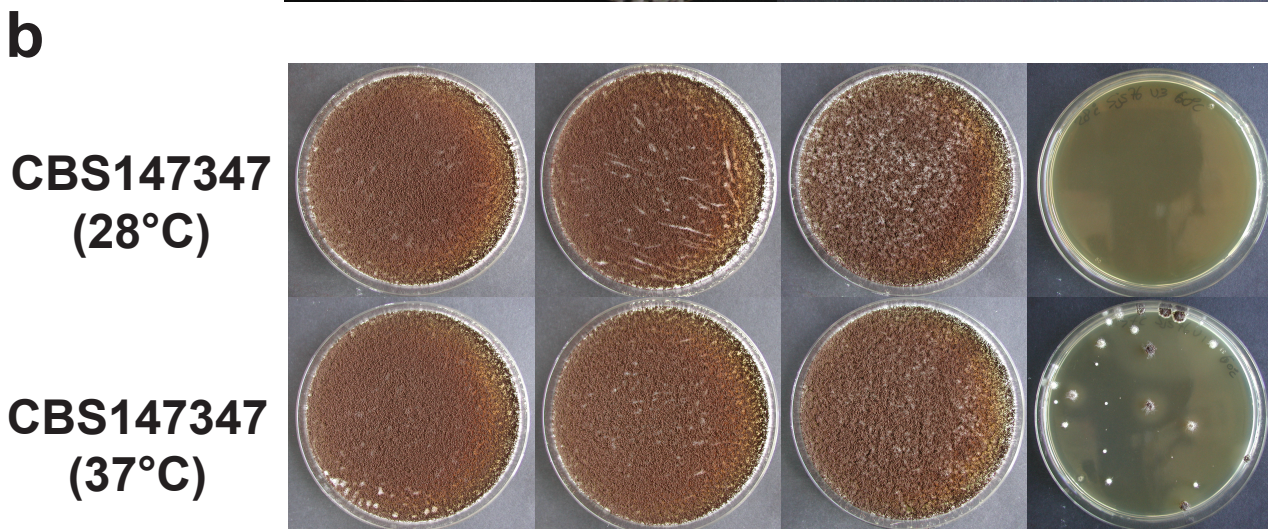

Supplement: Supplementary file 1 — Additional file 1: Figure S1 Cultivation temperature impacts heat resistance of wild-type A. niger strains CBS112.32 and CBS147347. Heat resistance was investigated using the heat treatment assay, in which conidia are harvested, diluted and subsequently heat treated for 10 minutes in a thermocycler after which 106 conidia are plated. Pictures were taken after 5 days of incubation at 28 °C. a, Heat resistance of wild-type strain A. niger CBS112.32. Conidial heat resistance increases when conidia were harvested from plates cultivated at 37 °C versus 28°C as seen by the colony forming units obtained after 10 minutes at 58 °C. b, Heat resistance of wild-type strain A. niger CBS147347. Conidial heat resistance increases when conidia were harvested from plates cultivated at 37 °C versus 28 °C as seen by the colony forming units obtained after 10 minutes at 60 °C. [file 40694_2023_168_MOESM1_ESM.pdf]

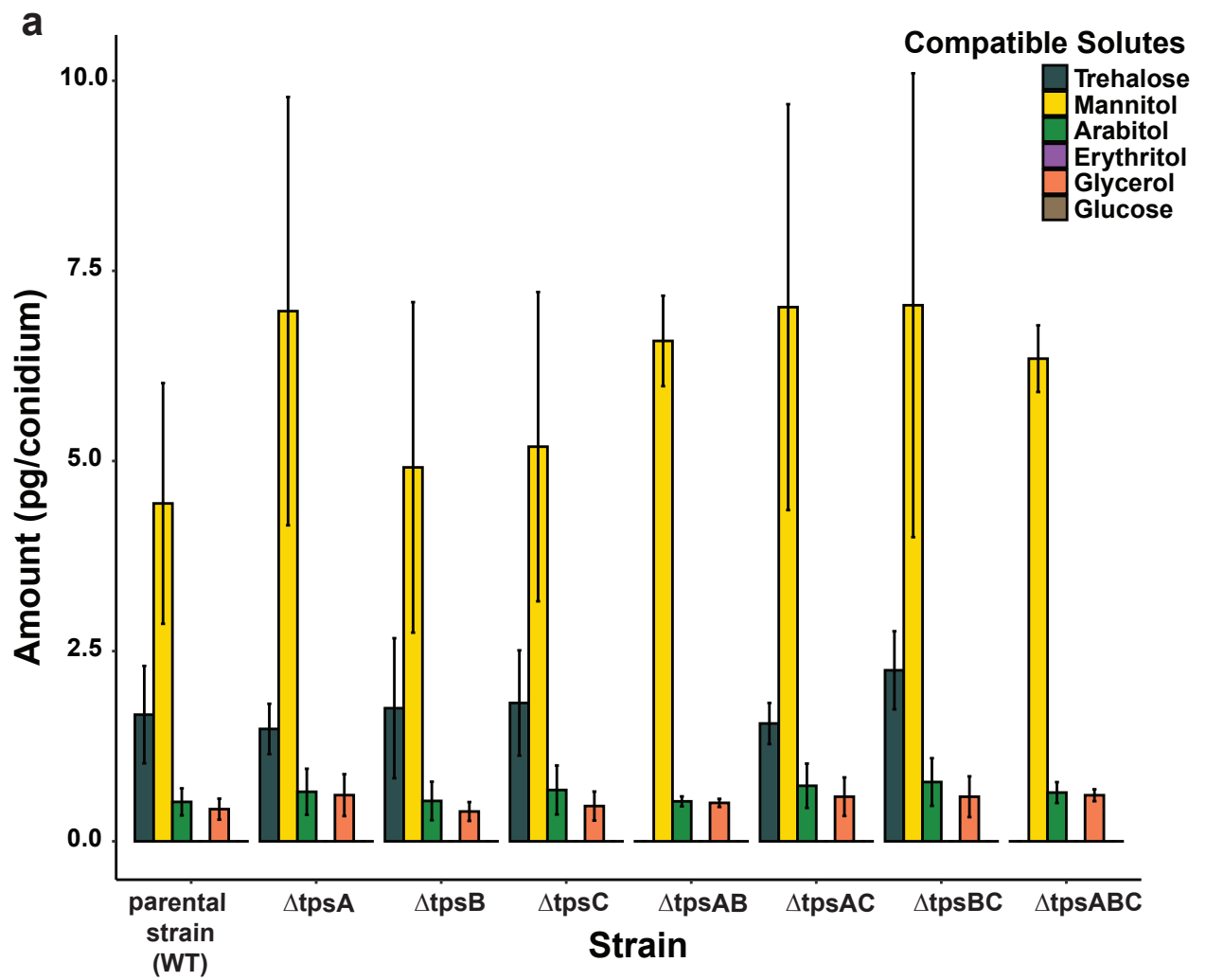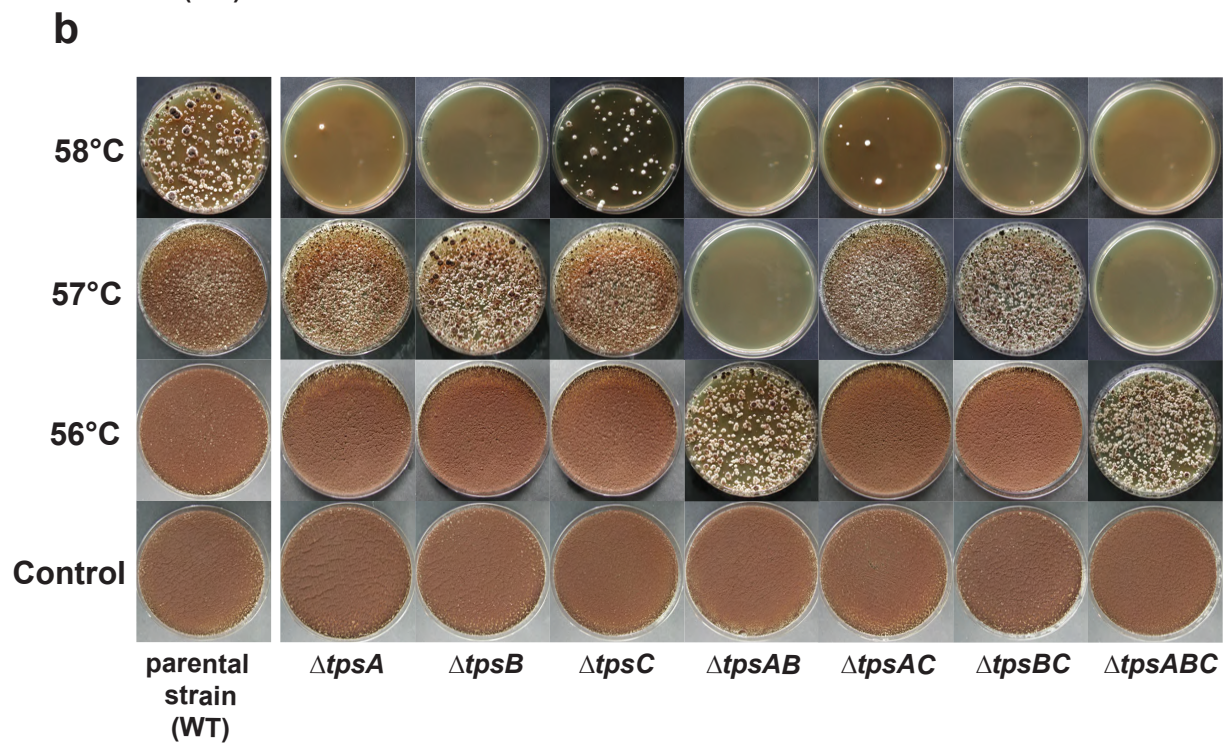

Supplement: Supplementary file 2 — Additional file 2: Fig. S2 Internal compatible solute composition and heat resistance of conidia from A. niger trehalose knock-out strains. Conidia were freshly harvested from MEA plates grown for 8 days at 28°C. Measurements were performed in biological triplicates. a, Internal compatible solute composition of conidia from trehalose knock-out strains as determined by HPLC analysis. Conidia from ΔtpsAB and ΔtpsABC strains were significantly impacted in their internal compatible solute composition, as they produced no measurable amount of trehalose. No significant increase in any other type of sugar was measured in these two strains. b, Heat treatments were applied for 10 minutes to 106 conidia. After heat treatments conidia were plated on plates containing MEA+0.05% triton x-100. Plates were grown for 5 days at 28°C after which the pictures were made as shown above. All conidia from knock-out strains lacking tps genes showed at least a slight decrease in heat resistance. However, the largest drop in heat resistance was observed in strains ΔtpsAB and ΔtpsABC. This drop in conidial heat resistance corresponds with the decrease and overall absence of trehalose inside these conidia. Overall, a clear link is seen between internal trehalose concentration and conidial heat resistance. [file 40694_2023_168_MOESM2_ESM.pdf]

**a**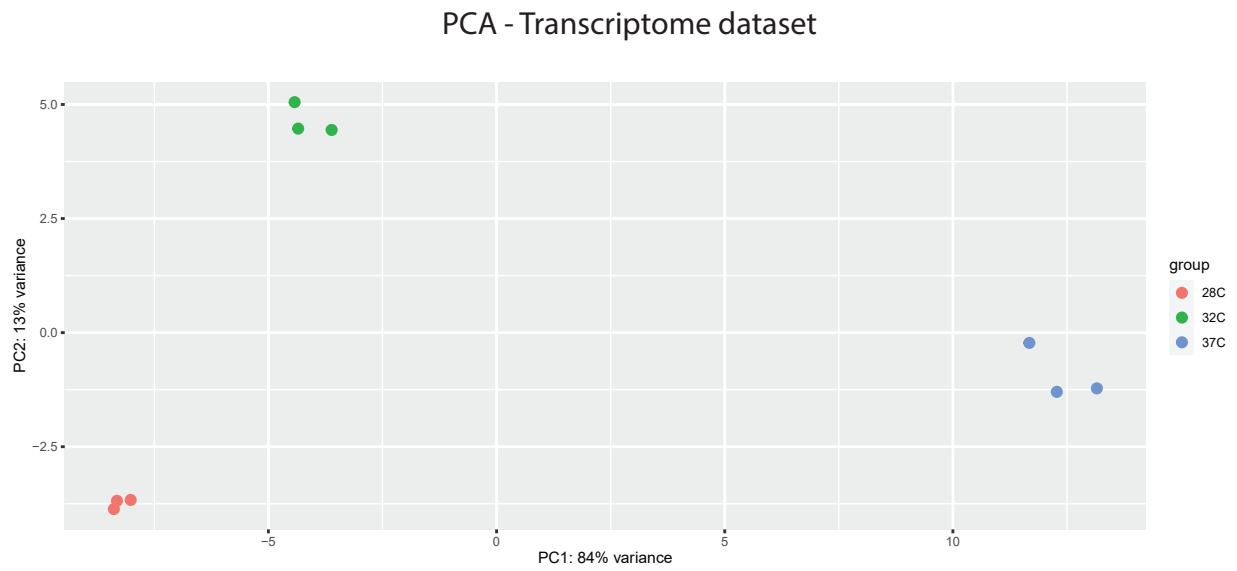**b**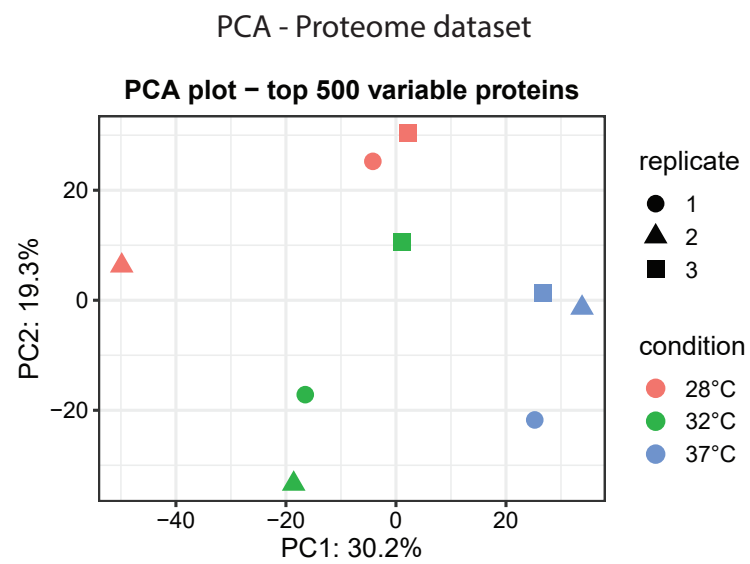

Supplement: Supplementary file 3 — Additional file 3: Figure S3. Heat shock results of strains complemented to have a wild type genotype. Conidia were freshly harvested from MEA plates grown for 8 days at 28°C. Measurements were performed in biological triplicates. Complemented strains were checked for two silent point mutations introduced into the genes (see Materials and Methods) to distinguish between the original wild type and the complemented strains. All knock-out strains were successfully complemented back to wild type genotype and their conidia show wild type levels of heat resistance. [file 40694_2023_168_MOESM3_ESM.pdf]

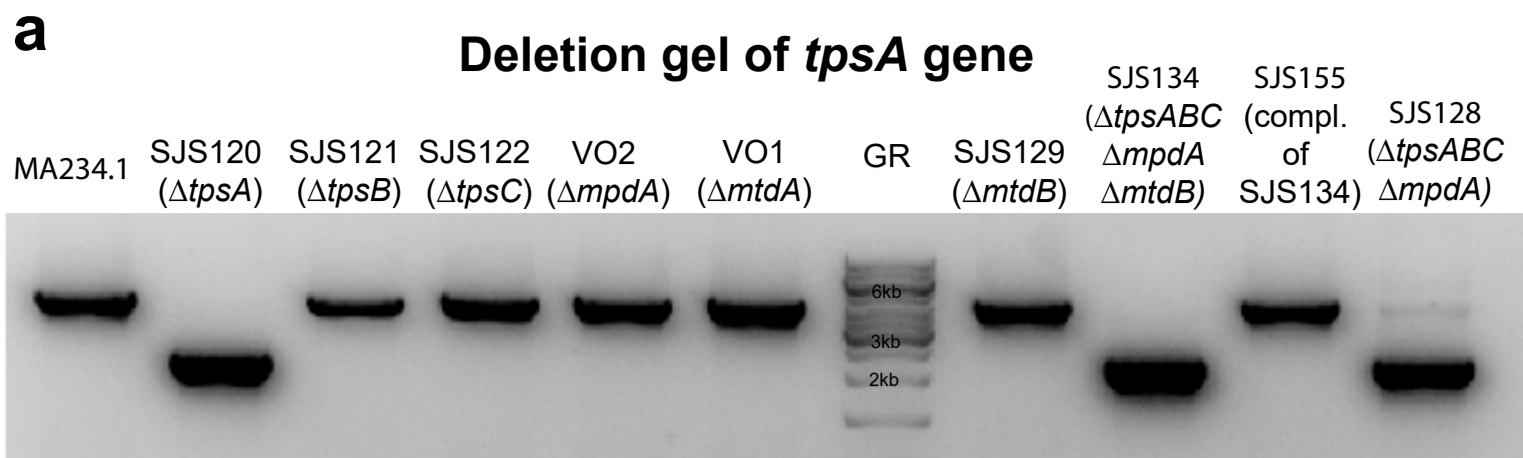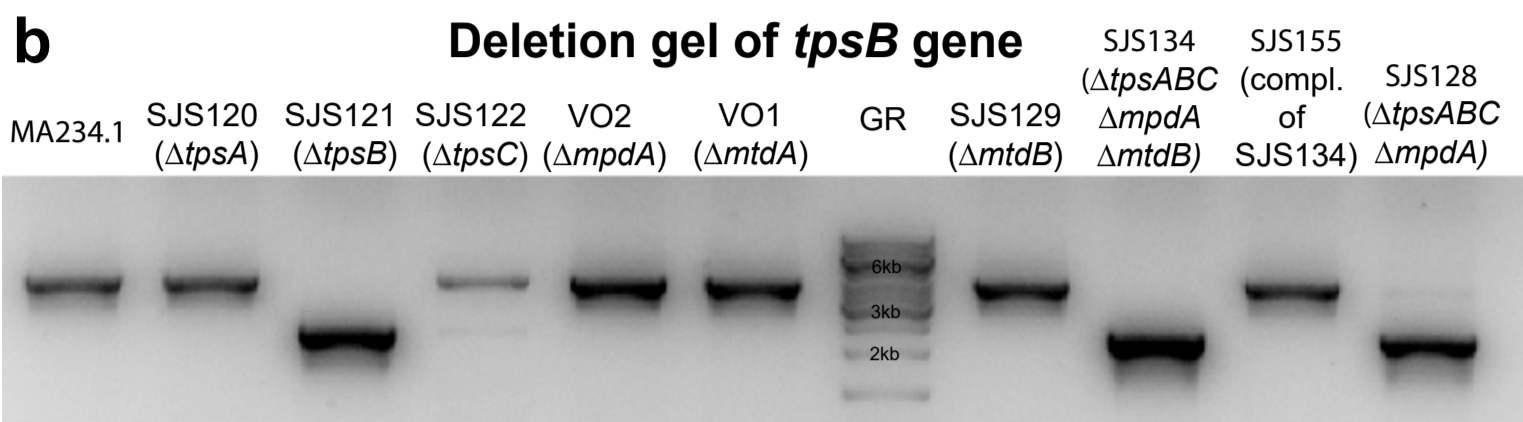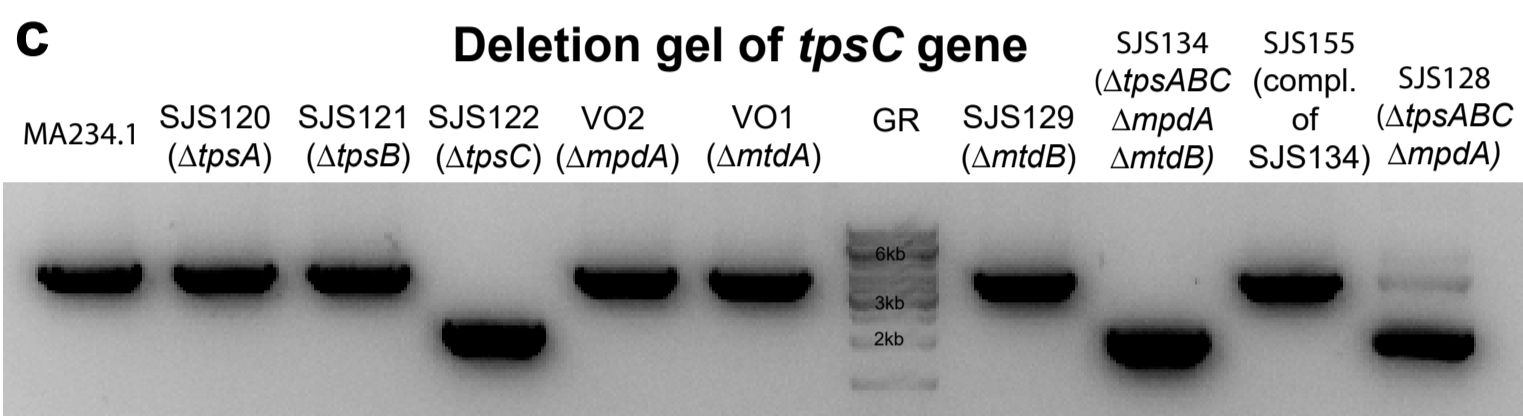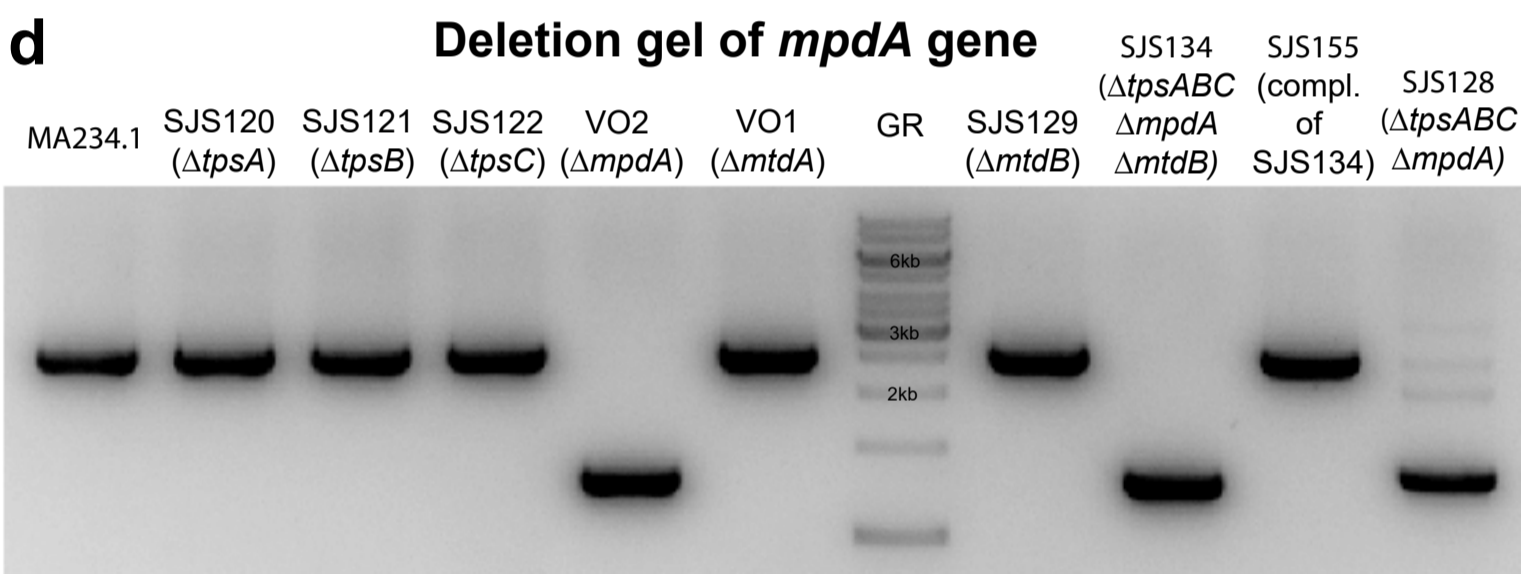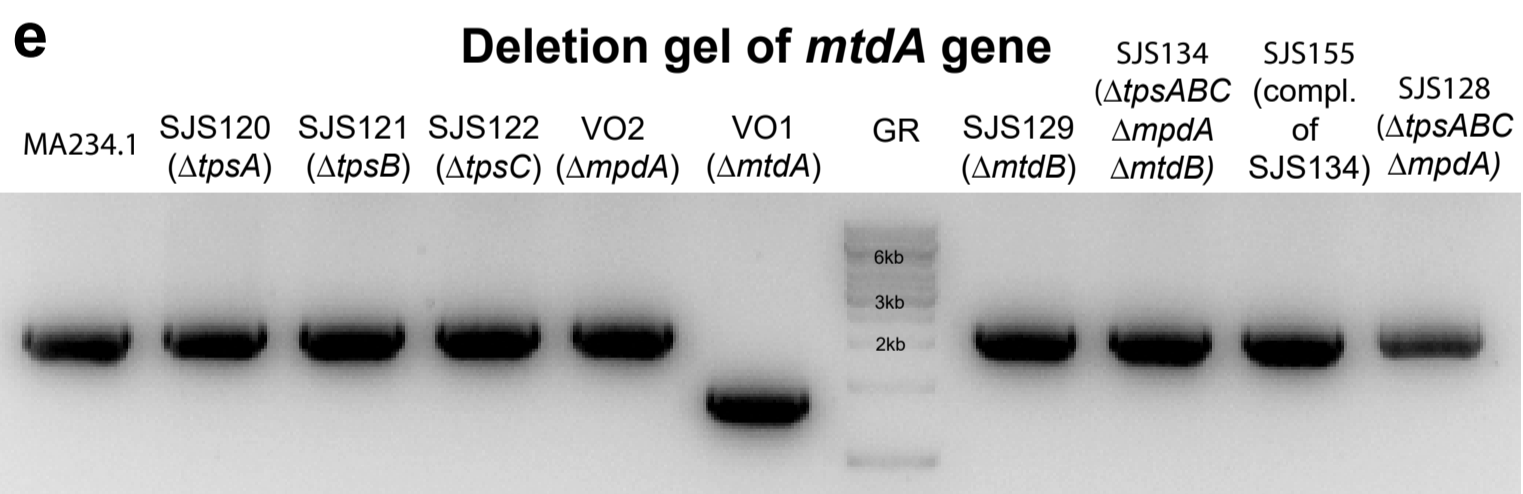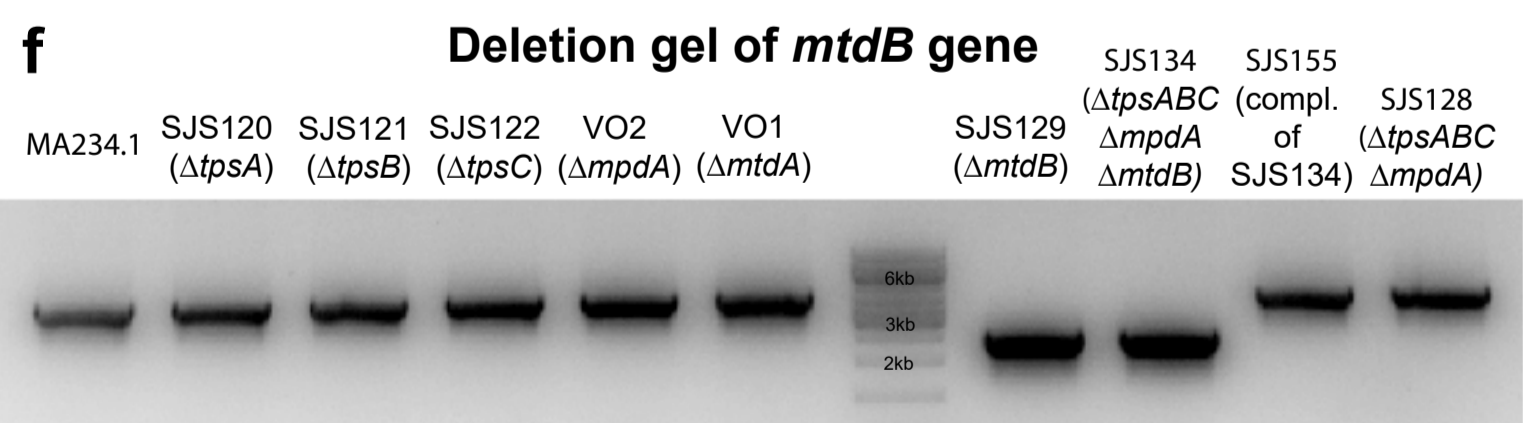

Supplement: Supplementary file 4 — Additional file 4: Figure S4 Principal component analysis (PCA) on the transcriptome and proteome datasets. The three cultivation conditions; 28 °C, 32 °C and 37 °C were compared. Most of the variance can be explained by the x-axis, of which the 37 °C condition is the largest contributing factor in both cases. a, PCA of the transcriptome data, 84% of the variance is found on the x-axis which is mostly due to the 37°C condition. b, PCA of the proteome data. Variance is not as large as in the transcriptome dataset, but most of it is due to the x-axis difference which is due to the 37°C condition. [file 40694_2023_168_MOESM4_ESM.pdf]

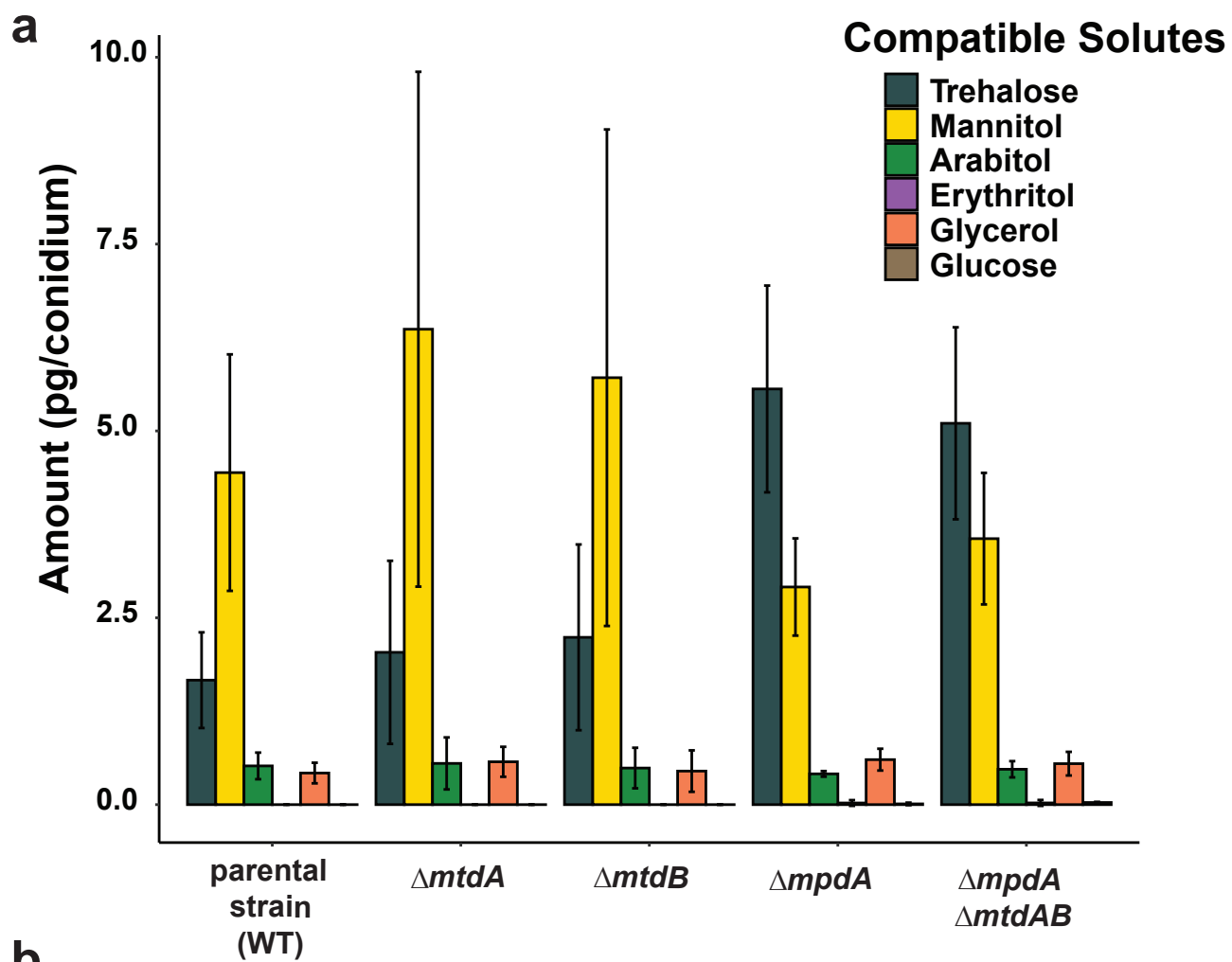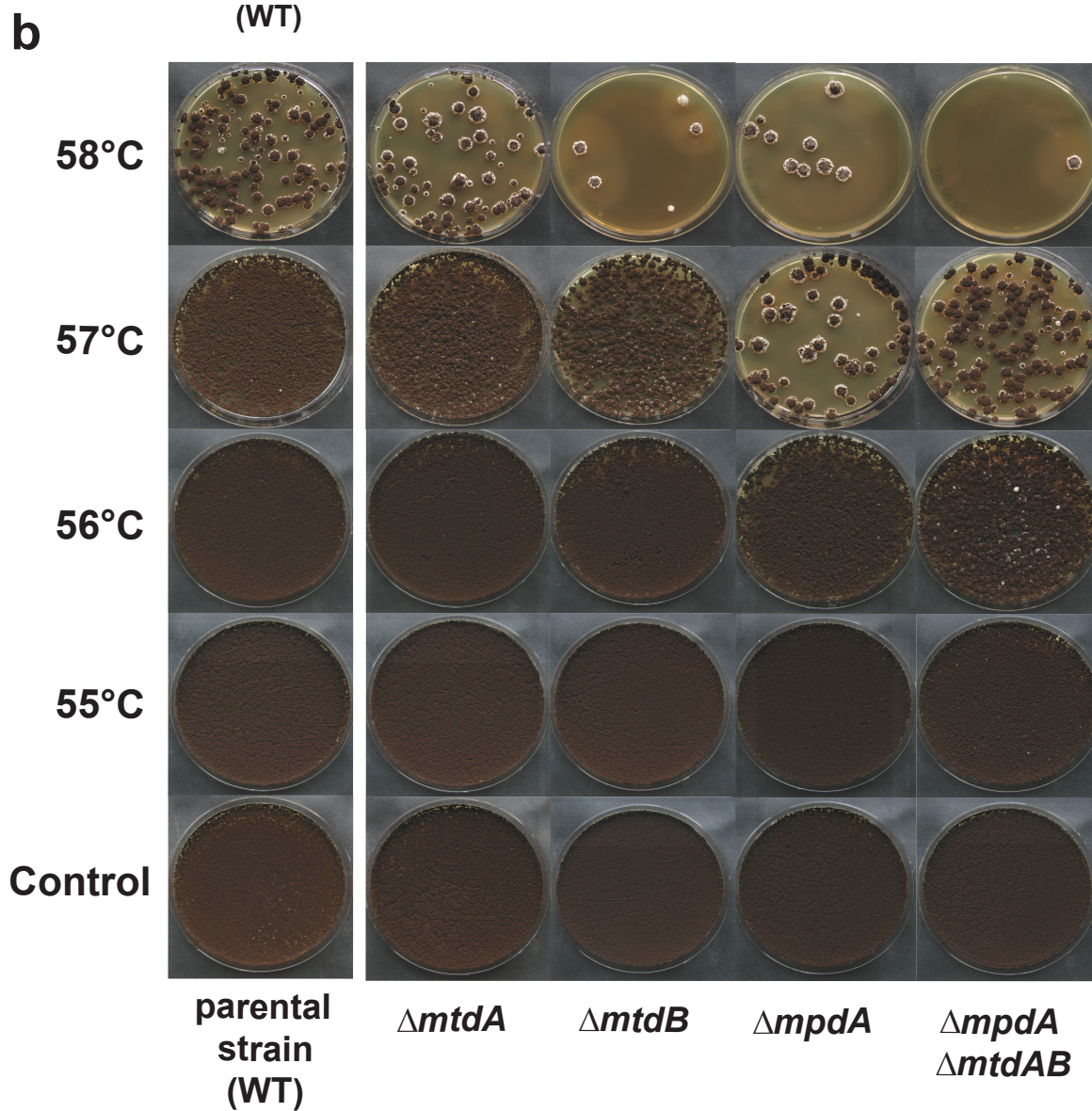

Supplement: Supplementary file 5 — Additional file 5: Fig. S5 Diagnostic PCR confirming deletions in knock-out strains. Each column on the gel represents an amplification product from the wild type strain MA234.1, a gene knock-out strain, or the GeneRuler ladder. a, Deletion of the tpsA gene. Amplification was done using diagnostic primers DIAG_tpsA_5'_fw and DIAG_tpsA_3'_rv. If the gene is present, a band size of 4118 bps is expected. If the gene is absent, a band size of 2189 bps is expected. b, Deletion of the tpsB gene. Amplification was done using diagnostic primers DIAG_tpsB_5'_fw and DIAG_tpsB_3'_rv. If the gene is present, a band size of 3994 bps is expected. If the gene is absent, a band size of 2281 bps is expected. c, Deletion of the tpsC gene. Amplification was done using diagnostic primers DIAG_tpsC_5'_fw and DIAG_tpsC_3'_rv. If the gene is present, a band size of 3894 bps is expected. If the gene is absent, a band size of 2196 bps is expected. d, Deletion of the mpdA gene. Amplification was done using diagnostic primers DIAG_mpdA_5'_fw and DIAG_mpdA_3'_rv. If the gene is present, a band size of 2499 bps is expected. If the gene is absent, a band size of 1345 bps is expected. e, Deletion of the mtdA gene. Amplification was done using diagnostic primers DIAG_mtdA_5'_fw and DIAG_mtdA_3'_rv. If the gene is present, a band size of 2176 bps is expected. If the gene is absent, a band size of 1387 bps is expected. f, Deletion of the mtdB gene. Amplification was done using diagnostic primers DIAG_MtdB_5'_fw and DIAG_MtdB_3'_rv. If the gene is present, a band size of 4233 bps is expected. If the gene is absent, a band size of 2347 bps is expected. [file 40694_2023_168_MOESM5_ESM.pdf]

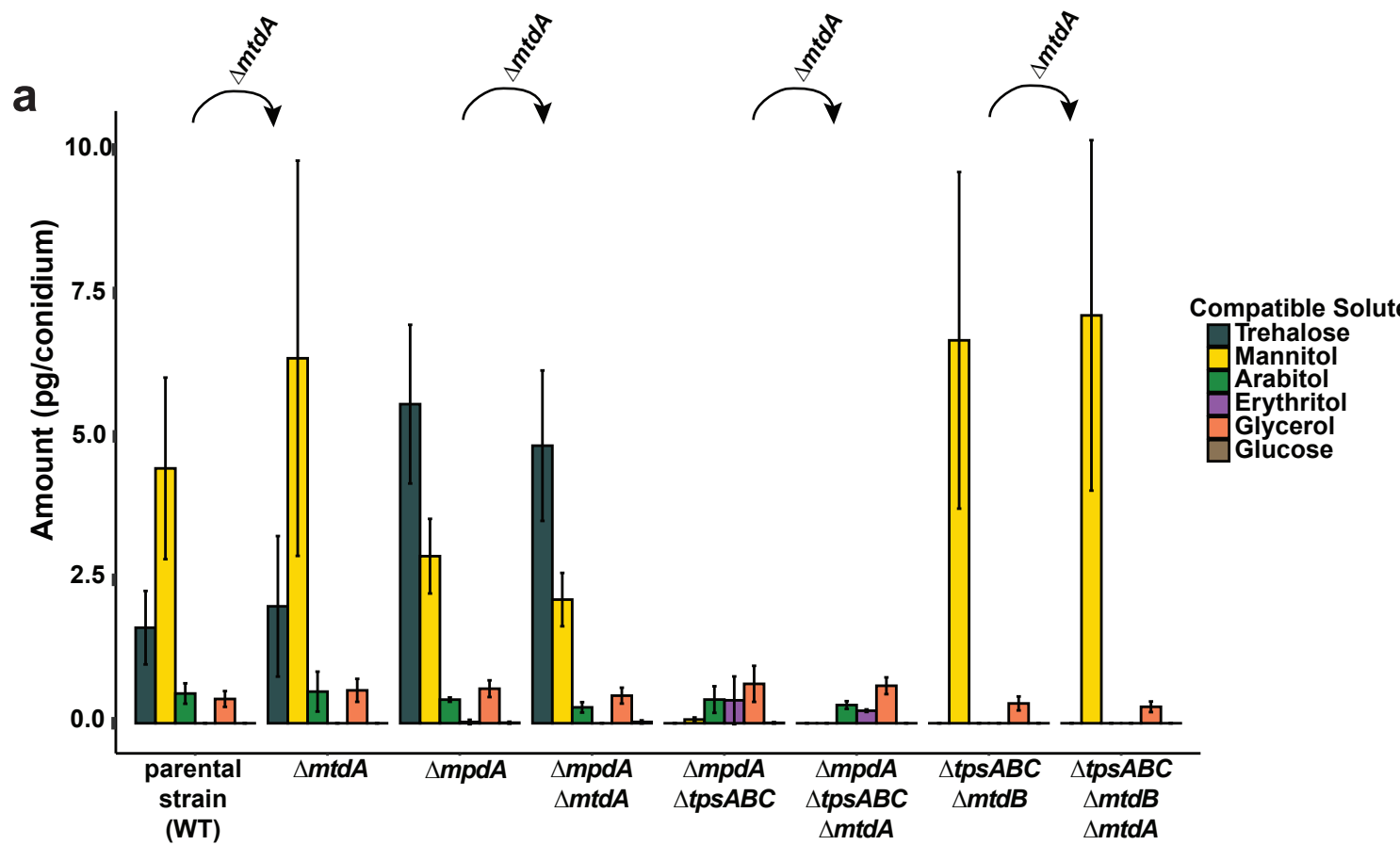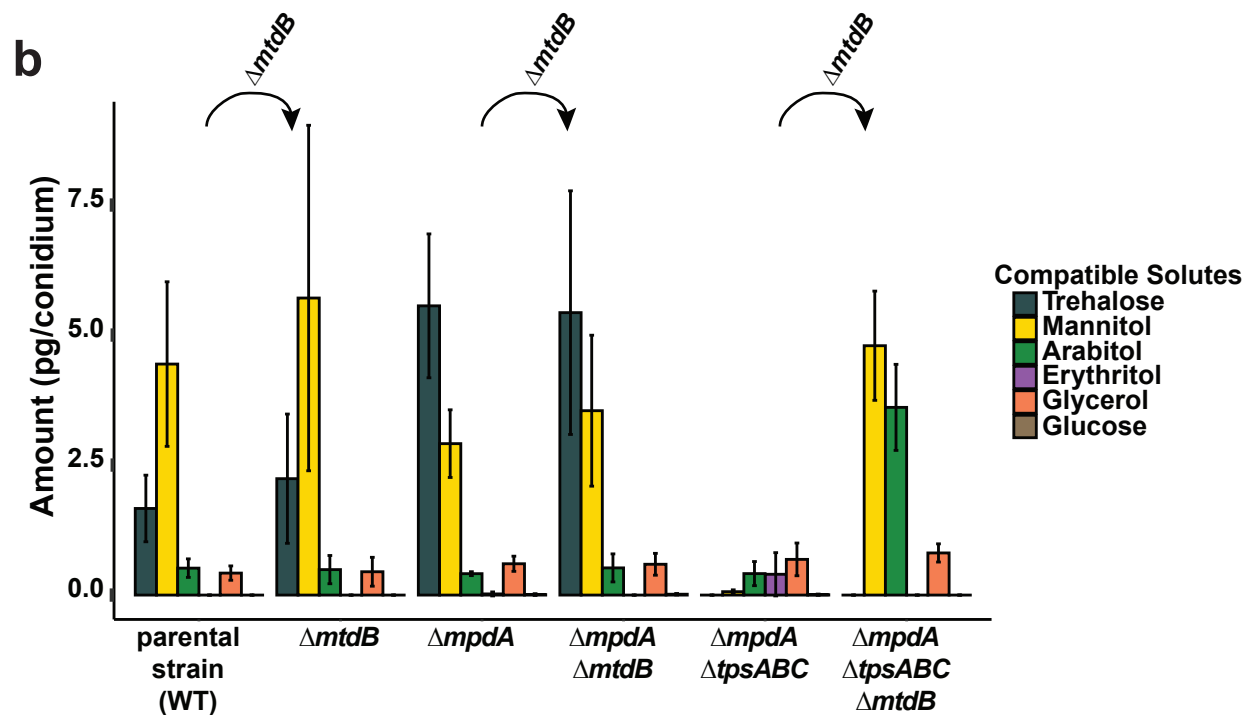

Supplement: Supplementary file 6 — Additional file 6: Fig. S6 Internal compatible solute composition and heat resistance of conidia from A. niger mannitol cycle knock-out strains. Conidia were freshly harvested from MEA plates grown for 8 days at 28°C. Measurements were performed in biological triplicates. a, Internal compatible solute composition of conidia from mannitol knock-out strains lacking genes involved in the mannitol cycle. Only the deletion of the mpdA gene resulted in a change of internal sugar composition inside conidia of A. niger. Strain ΔmpdA contains less mannitol and more trehalose as has been observed before (17). The internal compatible solute composition of the strain ΔmpdA, ΔmtdAB was the same as observed for ΔmpdA. As such, no effect of both mtdA or mtdB deletion on compatible solute composition of conidia was seen in these knock-out strains. b, Heat treatments were applied for 10 minutes to 106 conidia. After heat treatment, conidia were plated on plates containing MEA+0.05% triton x-100. Plates were grown for 5 days after which the pictures were made as shown above. The mpdA deletion has the largest effect on the heat resistance of A. niger conidia, as the number of observed CFUs is less than 100 when heat stress of 57°C is applied. The strain in which mpdA and both mannitol dehydrogenases mtdA and mtdB have been knocked out, is comparable in heat resistance to the ΔmpdA single knock-out strain, suggesting that mtdA and mtdB do not significantly contribute to the heat resistance of A. niger conidia. [file 40694_2023_168_MOESM6_ESM.pdf]

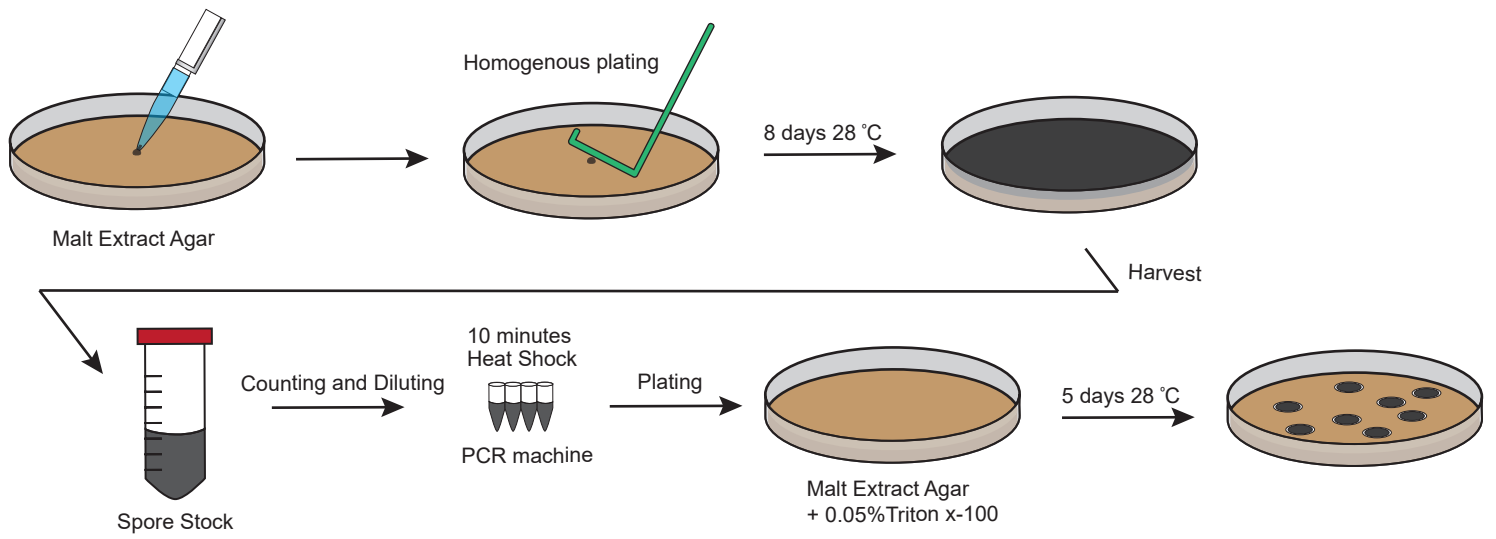

Supplement: Supplementary file 8 — Additional file 8: Fig. S8 Heat treatment assay protocol. All strains were plated homogenously and subsequently grown on MEA for 8 days at 28°C, unless noted otherwise. Conidia were harvested in PS buffer and subsequently counted using a Bio-Rad Automated Cell Counter. A total volume of 100 µl containing 1*106 conidia were heat treated per PCR tube in a thermocycler for 10 minutes. After heat treatment the 100 µl is plated homogenously on MEA + 0.05% Triton X-100 and grown for 5 days at 28°C after which CFUs are counted and pictures were taken. [file 40694_2023_168_MOESM8_ESM.pdf]
