# Supplementary material for: Compatible solutes determine the heat resistance of conidia
Source: Fungal Biol Biotechnol. 2023 Nov 13;10:21. doi: 10.1186/s40694-023-00168-9 (PMC10644514; doi:10.1186/s40694-023-00168-9)
Supplement: Supplementary file 10 — Additional file 10: Table S2. The 60 proteins significantly more or less present in conidia cultivated at 37°C. Positive values (green) in Log2 fold changes found significant (p < 0.05) show upregulation in conidia cultivated at 37°C, whereas negative values (red) show downregulation in conidia cultivated at 37°C. Descriptions are based on EuKaryotic Orthologous Groups (KOG) found as part of MycoCosm on the JGI website 68. All descriptions are putative and solely based on homology. The baseMean DESeq2 values represent the average of normalized counts and the LFQ intensities represent quantified proteome data based on peptides found (higher values = more protein present). Normalization, Log2FC and their significance were calculated with the DESeq2 package in R for the transcriptome data and the DEP package in R for the proteome data. [file 40694_2023_168_MOESM10_ESM.docx]

**Table S2. The 60 proteins significantly more or less present in conidia cultivated at 37°C.** Positive values (green) in Log2 fold changes found significant (p < 0.05) show upregulation in conidia cultivated at 37**°**C, whereas negative values (red) show downregulation in conidia cultivated at 37**°**C. Descriptions are based on EuKaryotic Orthologous Groups (KOG) found as part of MycoCosm on the JGI website ^68^. All descriptions are putative and solely based on homology. The baseMean DESeq2 values represent the average of normalized counts and the LFQ intensities represent quantified proteome data based on peptides found (higher values = more protein present). Normalization, Log2FC and their significance were calculated with the DESeq2 package in R for the transcriptome data and the DEP package in R for the proteome data.

|  | |  | |  | | **Transcriptome** | | | | **Proteome** | | | |  |
| --- | --- | --- | --- | --- | --- | --- | --- | --- | --- | --- | --- | --- | --- | --- |
| **NRRL3-number** | **An-number** | | **Description** | | **baseMean DESeq2** | | **Log_2_FC_transcriptome** | **p-value** | **Average_LFQintensity_28C** | | **Average_LFQintensity_37C** | **log_2_FC_proteome** | **p-value** | |
| NRRL3_10215 | An18g00600 | | Molecular chaperone (small heat-shock protein Hsp26/Hsp42) | | 12309 | | 2.43 | 2.44E-44 | 5900 | | 1178113 | 7.5 | 2.6E-13 | |
| NRRL3_04002 | An15g05410 | | Molecular chaperone (small heat-shock protein Hsp26/Hsp42) | | 1759 | | 4.60 | 1.14E-65 | 0 | | 130312 | 7.01 | 2.2E-08 | |
| NRRL3_09378 | An11g09460 | | Sorting nexin SNX11 | | 606 | | 0.17 | 0.593362 | 0 | | 97338 | 5.94 | 4.9E-04 | |
| NRRL3_10516 | An18g04270 | | Parvulin-like peptidyl-prolyl cis-trans isomerase | | 237 | | -0.13 | 0.762198 | 0 | | 46732 | 5.85 | 1.6E-03 | |
| NRRL3_11626 | An06g01530 | | Glucan 1,3-beta-glucosidase | | 33 | | -0.04 | 0.940552 | 2858 | | 196177 | 5.64 | 1.3E-06 | |
| NRRL3_05700 | An02g07130 | | Mitochondrial large subunit ribosomal protein (Img2) | | 499 | | 0.94 | 5.76E-07 | 0 | | 149847 | 5.63 | 3.2E-04 | |
| NRRL3_11083 | An08g04410 | | NADH-ubiquinone oxidoreductase subunit | | 568 | | -0.50 | 0.147736 | 0 | | 125943 | 5.3 | 2.7E-03 | |
| NRRL3_03693 | An15g01410 | | Possible oxidoreductase | | 460 | | 0.17 | 0.626468 | 0 | | 66393 | 5.28 | 1.4E-06 | |
| NRRL3_00318 | An09g03890 | | Glyoxylate/hydroxypyruvate reductase | | 361 | | -0.37 | 0.213897 | 0 | | 99540 | 5.27 | 2.5E-07 | |
| NRRL3_11707 | An06g00650 An06g00660 | | Oxoprolinase | | 1303 | | 0.14 | 0.652759 | 218194 | | 349483 | 5.16 | 2.0E-07 | |
| NRRL3_06627 | An16g09040 | | N-acetyl-glucosamine-6-phosphate deacetylase | | 1674 | | -0.13 | 0.61331 | 4435 | | 115895 | 5.05 | 1.1E-03 | |
| NRRL3_08471 | An03g04500 | | Nucleoside diphosphate-sugar hydrolase of the MutT (NUDIX) family | | 286 | | -0.24 | 0.5011 | 2028 | | 66817 | 4.85 | 3.7E-03 | |
| NRRL3_07783 | An04g05750 | | hypothetical protein with signal peptide for secretion | | 460 | | -0.46 | 0.089005 | 0 | | 55409 | 4.62 | 1.2E-02 | |
| NRRL3_09219 | An11g11260 | | Protein-L-isoaspartate(D-aspartate) O-methyltransferase | | 3461 | | 0.34 | 0.188724 | 11574 | | 171360 | 4.57 | 3.6E-02 | |
| NRRL3_02034 | An01g05040 | | dUTPase | | 85 | | -2.36 | 0.002346 | 0 | | 30726 | 4.56 | 1.8E-02 | |
| NRRL3_07644 | An04g07530 | | G protein-coupled receptor | | 1954 | | 0.07 | 0.877749 | 58759 | | 1304167 | 4.5 | 8.7E-03 | |
| NRRL3_05517 | An02g09030 | | Nucleolar GTPase/ATPase p130 | | 27504 | | -0.22 | 0.05141 | 0 | | 58260 | 4.44 | 2.1E-02 | |
| NRRL3_00602 | An14g00300 | | 1-Acyl dihydroxyacetone phosphate reductase and related dehydrogenases | | 115 | | -0.08 | 0.857974 | 0 | | 40197 | 4.38 | 2.7E-02 | |
| NRRL3_06470 | An17g00880 | | Damage-control phosphatase ARMT1-like domain | | 284 | | 0.14 | 0.575414 | 0 | | 83084 | 4.23 | 8.9E-05 | |
| NRRL3_02586 | An01g11680 | | cis-muconate cyclase | | 170 | | 0.03 | 0.940182 | 5144 | | 99614 | 4.23 | 3.2E-03 | |
| NRRL3_00413 | An09g05140 | | Saccharopine dehydrogenase NADP binding domain | | 83 | | -2.28 | 2.29E-12 | 0 | | 26898 | 4.17 | 7.3E-06 | |
| NRRL3_11096 | An08g04540 | | Putative cyclase | | 83 | | -0.26 | 0.561376 | 4433 | | 80027 | 4.05 | 1.5E-02 | |
| NRRL3_05056 | An02g14900 An02g14910 | | Ubiquitin activating E1 enzyme-like protein | | 1866 | | -0.14 | 0.415603 | #N/A | | #N/A | 3.56 | 3.8E-02 | |
| NRRL3_04347 | An07g01530 | | GatB domain | | 63 | | -0.19 | 0.666369 | 26075 | | 258020 | 3.32 | 1.1E-02 | |
| NRRL3_04490 | An07g03340 | | Fungal hydrophobin *hyp1* | | 293 | | 0.00 | 0.995224 | 539587 | | 5093167 | 3.26 | 2.3E-02 | |
| NRRL3_09330 | An11g09920 | | Apoptosis-related protein/predicted DNA-binding protein | | 439 | | -0.21 | 0.521993 | 50923 | | 374457 | 2.86 | 4.1E-02 | |
| NRRL3_03532 | An05g00140 | | Signal recognition particle receptor, beta subunit (small G protein superfamily) | | 682 | | -0.28 | 0.114883 | 243067 | | 67113 | -2.08 | 4.2E-02 | |
| NRRL3_02666 | An01g12550 | | Mannosyl-oligosaccharide alpha-1,2-mannosidase and related glycosyl hydrolases | | 3984 | | -0.66 | 0.149562 | 380070 | | 69265 | -2.53 | 5.9E-03 | |
| NRRL3_00410 | An09g05110 | | Acyl-CoA synthetase | | 23559 | | -1.57 | 8.83E-05 | 256357 | | 43786 | -2.73 | 9.1E-03 | |
| NRRL3_02657 | An01g12450 | | Chitinase | | 2316 | | -2.40 | 2.9E-05 | 1256357 | | 176317 | -2.83 | 9.6E-03 | |
| NRRL3_06237 | An02g00210 | | Non-ribosomal peptide synthetase/alpha-aminoadipate reductase and related enzymes | | 2397 | | -0.42 | 0.008291 | 101361 | | 16332 | -2.84 | 4.2E-02 | |
| NRRL3_03373 | An12g04700 | | Dipeptidyl aminopeptidase | | 154 | | -0.79 | 0.069459 | 231517 | | 34358 | -3.04 | 5.2E-03 | |
| NRRL3_10599 | An18g05500 | | Ceramidase | | 1652 | | -0.30 | 0.354418 | 166843 | | 24713 | -3.05 | 3.3E-02 | |
| NRRL3_04237 | An07g00110 | | Beta-lactamase | | 406 | | -0.86 | 0.001611 | 905467 | | 122393 | -3.21 | 7.8E-03 | |
| NRRL3_00071 | An09g00810 | | Zinc-binding oxidoreductase | | 303 | | -1.40 | 2.81E-08 | 176773 | | 20027 | -3.31 | 2.9E-03 | |
| NRRL3_00279 | An09g03450 | | D-ribulose-5-phosphate 3-epimerase | | 922 | | -0.57 | 0.005181 | 16737 | | 0 | -3.49 | 9.2E-03 | |
| NRRL3_06024 | An02g02930 | | ribose-5-phosphate isomerase | | 2280 | | -0.30 | 0.294115 | 701343 | | 80009 | -3.56 | 1.6E-02 | |
| NRRL3_10970 | An08g03090 | | Calcium transporting ATPase | | 7420 | | 0.15 | 0.457215 | 167843 | | 19365 | -3.67 | 4.6E-03 | |
| NRRL3_06352 | An10g00800 | | Purine nucleoside permease (NUP) | | 32 | | -1.06 | 0.082256 | 325673 | | 25999 | -3.69 | 2.0E-04 | |
| NRRL3_02923 | An12g10470 | | cyclin-dependent kinase | | 7273 | | -0.40 | 0.166186 | 1360567 | | 119020 | -3.84 | 3.4E-03 | |
| NRRL3_11047 | An08g03960 | | Putative cargo transport protein ERV29 | | 719 | | -0.39 | 0.142135 | 129678 | | 7651 | -4.15 | 2.1E-02 | |
| NRRL3_02139 | An01g06310 | | hypothetical protein with DUF1793 domain | | 98 | | 0.38 | 0.369748 | 52896 | | 0 | -4.36 | 3.7E-03 | |
| NRRL3_03251 | An12g06060 | | hypothetical protein with YrdC-like domain | | 301 | | -1.87 | 6.26E-11 | 128167 | | 6207 | -4.41 | 2.2E-06 | |
| NRRL3_03449 | An12g03850 | | ATP-dependent RNA helicase | | 1153 | | 1.15 | 1.93E-12 | 42187 | | 0 | -4.42 | 9.7E-04 | |
| NRRL3_04236 | An07g00100 | | Amidase | | 2983 | | -2.77 | 2.81E-50 | 50961 | | 0 | -4.5 | 4.8E-04 | |
| NRRL3_10468 | An18g03780 | | Aminopeptidases of the M20 family | | 672 | | -0.13 | 0.662217 | 337473 | | 14022 | -4.58 | 8.0E-04 | |
| NRRL3_06750 | An16g07450 | | Translation initiation factor 2C (eIF-2C) and related proteins | | 1599 | | -1.41 | 1.47E-10 | 239267 | | 9344 | -4.63 | 3.8E-08 | |
| NRRL3_03138 | An12g07570 | | Synaptobrevin/VAMP-like protein | | 2175 | | 0.97 | 1.39E-32 | 120507 | | 2519 | -4.73 | 1.7E-02 | |
| NRRL3_06942 | An16g04640 | | Predicted membrane protein | | 318 | | 0.00 | 0.998817 | 53285 | | 0 | -4.73 | 2.3E-02 | |
| NRRL3_02536 | An01g11100 | | Predicted membrane protein | | 8115 | | 0.33 | 0.011983 | 40871 | | 0 | -4.77 | 6.9E-03 | |
| NRRL3_03346 | An12g04950 | | Mitochondrial F1F0-ATP synthase, subunit epsilon/ATP15 | | 937 | | -0.04 | 0.940168 | 54565 | | 0 | -4.84 | 2.2E-02 | |
| NRRL3_08244 | An04g00150 | | Glutaredoxin-related protein | | 502 | | -0.06 | 0.8883 | 85849 | | 6223 | -4.85 | 2.4E-02 | |
| NRRL3_07734 | An04g06310 | | hypothetical protein with signal peptide for secretion | | 1476 | | -0.45 | 0.000469 | 61747 | | 0 | -5.06 | 4.3E-02 | |
| NRRL3_09480 | An11g08250 | | Glutamate decarboxylase and related proteins | | 185 | | -0.90 | 0.016693 | 290377 | | 6787 | -5.46 | 1.5E-05 | |
| NRRL3_11110 | An08g04690 | | Dehydrogenases with different specificities (related to short-chain alcohol dehydrogenases) | | 141 | | -3.62 | 2.02E-09 | 192914 | | 3916 | -5.59 | 4.6E-04 | |
| NRRL3_04169 | An15g07370 | | Chitinase | | 26 | | -0.35 | 0.414464 | 111428 | | 0 | -5.94 | 1.5E-06 | |
| NRRL3_03454 | An12g03760 | | hypothetical protein | | 161 | | 2.41 | 0.000117 | 1259540 | | 21374 | -6.04 | 2.3E-05 | |
| NRRL3_03951 | An15g04790 | | Fungal specific transcription factor domain containing protein | | 372 | | 0.35 | 0.135284 | 165789 | | 0 | -6.26 | 2.6E-03 | |
| NRRL3_10314 | An18g01890 | | hypothetical protein | | 3670 | | -1.98 | 2.06E-12 | 1777000 | | 23598 | -6.44 | 2.6E-13 | |
| NRRL3_04228 | An07g00020 | | alpha/beta hydrolase | | 206 | | 0.35 | 0.394227 | 137507 | | 0 | -7.7 | 2.1E-02 | |
