# Supplementary material for: Compatible solutes determine the heat resistance of conidia
Source: Fungal Biol Biotechnol. 2023 Nov 13;10:21. doi: 10.1186/s40694-023-00168-9 (PMC10644514; doi:10.1186/s40694-023-00168-9)
Supplement: Supplementary file 12 — Additional file 12: Table S4. List of strains used in this study. [file 40694_2023_168_MOESM12_ESM.docx]

**Table S3. List of strains used in this study**

| Name | Genotype | Parental strain | Reference | Extra info strain |
| --- | --- | --- | --- | --- |
| N402 | *cspA1, amdS*- | - | (67) | Wild type |
| MA234.1 | *cspA1,* Δ*kusA::DR*-*amdS*-*DR* | N402 | (55) |  |
| SJS143 | Δ*NRRL3_01479; ‘*Δ*dprA’* | MA234.1 | This study | Strain lacking a gene coding for a putative dehydrin-like protein (45) |
| SJS144 | Δ*NRRL3_01479; ‘*Δ*dprB’* | MA234.1 | This study | Strain lacking a gene coding for a putative dehydrin-like protein (45) |
| SJS145 | Δ*NRRL3_01479,* Δ*NRRL3_01479; ‘*Δ*dprAB’* | MA234.1 | This study | Strain lacking both putative dehydrin-like proteins (45) |
| SJS146 | Δ*NRRL3_05684; ‘*Δ*LEA3-like’* | MA234.1 | This study | Strain lacking a LEA3-like protein thought to be involved in the cell stress response (26, 68) |
| SJS147.2 | Δ*NRRL3_02511, ‘*Δ*conJ’* | MA234.1 | This study | Strain lacking a homologue of *conJ* as described in *A. nidulans* involved in stress response (46) |
| JZ2.1 | Δ*NRRL3_11620; ‘*Δ*hsp9/12’* | MA234.1 | This study | Strain lacking putative *hsp12* homologue, important for plasma membrane stability in *S. cerevisiae* (47, 48) |
| JZ3.1 | Δ*NRRL3_02725; ‘*Δ*hsp104’* | MA234.1 | This study | Strain lacking putative *hsp104* homologue, important for heat resistance in *S. cerevisiae* (49, 50, 69) |
| SJS123 | *cspA1,* Δ*kusA::DR*-*amdS*-*DR,* Δ*tpsAB* | SJS121 | This study |  |
| SJS124 | *cspA1,* Δ*kusA::DR*-*amdS*-*DR,* Δ*tpsAC* | MA234.1 | This study |  |
| SJS125 | *cspA1,* Δ*kusA::DR*-*amdS*-*DR,* Δ*tpsBC* | SJS121 | This study |  |
| SJS126 | *cspA1,* Δ*kusA::DR*-*amdS*-*DR,* Δ*tpsABC* | SJS121 | This study |  |
| SJS127 | *cspA1,* Δ*kusA::DR*-*amdS*-*DR,* Δ*mpdA,* Δ*tpsAB* | SJS123 | This study |  |
| SJS128 | *cspA1,* Δ*kusA::DR*-*amdS*-*DR,* Δ*mpdA,* Δ*tpsABC* | SJS126 | This study |  |
| SJS129 | *cspA1,* Δ*kusA::DR*-*amdS*-*DR,* Δ*mtdB* | MA234.1 | This study |  |
| SJS130 | *cspA1,* Δ*kusA::DR*-*amdS*-*DR,* Δ*mtdB,* Δ*mpdA* | VO2 | This study |  |
| SJS131 | *cspA1,* Δ*kusA::DR*-*amdS*-*DR,* Δ*mtdB,* Δ*tpsAB* | SJS123 | This study |  |
| SJS132 | *cspA1,* Δ*kusA::DR*-*amdS*-*DR,* Δ*mtdB,* Δ*tpsABC* | SJS126 | This study |  |
| SJS133 | *cspA1,* Δ*kusA::DR*-*amdS*-*DR,* Δ*mtdB,* Δ*mpdA,* Δ*tpsAB* | SJS127 | This study |  |
| SJS134 | *cspA1,* Δ*kusA::DR*-*amdS*-*DR,* Δ*mtdB,* Δ*mpdA,* Δ*tpsABC* | SJS128 | This study |  |
| SJS135 | *cspA1,* Δ*kusA::DR*-*amdS*-*DR,* Δ*tpsAB,* Δ*mtdA* | SJS123 | This study |  |
| SJS136 | *cspA1,* Δ*kusA::DR*-*amdS*-*DR,* Δ*tpsABC,* Δ*mtdA* | SJS126 | This study |  |
| SJS137 | *cspA1,* Δ*kusA::DR*-*amdS*-*DR* Δ*tpsABC,* Δ*mpdA,* Δ*mtdA* | SJS128 | This study |  |
| SJS138 | *cspA1,* Δ*kusA::DR*-*amdS*-*DR,* Δ*mtdAB* | SJS129 | This study |  |
| SJS139 | *cspA1,* Δ*kusA::DR*-*amdS*-*DR,* Δ*mtdAB,* Δ*mpdA* | SJS130 | This study |  |
| SJS141 | *cspA1,* Δ*kusA::DR*-*amdS*-*DR,* Δ*mtdAB,* Δ*tpsABC* | SJS132 | This study |  |
| SJS142 | *cspA1,* Δ*kusA::DR*-*amdS*-*DR,* Δ*mtdAB,* Δ*mpdA,* Δ*tpsABC* | SJS134 | This study |  |
| SJS149.2 | *cspA1,* Δ*kusA::DR*-*amdS*-*DR*, *mpdA* T669A, T672G | VO2 | This study |  |
| SJS152.3 | *cspA1, ΔkusA::DR*-*amdS*-*DR, tpsC* T93A, A96T | SJS126 | This study |  |
| SJS153 | *cspA1,* Δ*kusA::DR*-*amdS*-*DR, tpsA* T258C, G273A | SJS128 | This study |  |
| SJS154 | *cspA1,* Δ*kusA::DR*-*amdS*-*DR, tpsB* G267A, G270A | SJS132 | This study |  |
| SJS155 | *cspA1,* Δ*kusA::DR*-*amdS*-*DR, tpsA* T258C, G273A, *tpsB* G267A, G270A | SJS134 | This study |  |
| SJS156 | *cspA1,* Δ*kusA::DR*-*amdS*-*DR,* Δ*mpdA,* Δ*tpsABC* | SJS134 | This study |  |
| CBS112.32 | Wild type | - | (70) | Wild isolate |
| CSB147347 | Wild type | - | (70) | Wild isolate |
