# Supplementary material for: Compatible solutes determine the heat resistance of conidia
Source: Fungal Biol Biotechnol. 2023 Nov 13;10:21. doi: 10.1186/s40694-023-00168-9 (PMC10644514; doi:10.1186/s40694-023-00168-9)
Supplement: Supplementary file 13 — Additional file 13: Table S5. Colony forming units of A. niger conidia from knock-out strains plated on MEA. [file 40694_2023_168_MOESM13_ESM.docx]

**Table S4. Colony forming units of *A. niger* conidia from knock-out strains plated on MEA.**

| Strain | Average (CFUs) | Stdev (CFUs) |
| --- | --- | --- |
| Wild type | 107 | ±19 |
| Δ*tpsABC* | 107 | ±19 |
| Δ*mpdA,* Δ*tpsABC* | 88 | ±8 |
| Δ*mtdB,* Δ*tpsABC* | 92 | ±11 |
| Δ*mpdA,* Δ*mtdB,* Δ*tpsABC* | 73 | ±17 |

Conidia were counted using a Bio-Rad TC20™ automated cell counter, diluted until 100 conidia/100µl and subsequently confluently plated. This experiment was done in biological triplicates. No significantly lower amount (p<0.05 student t-test) of colonies were formed when conidia from knock-out strains were plated and compared to wild-type conidia of strain N402.
