# Supplementary material for: Compatible solutes determine the heat resistance of conidia
Source: Fungal Biol Biotechnol. 2023 Nov 13;10:21. doi: 10.1186/s40694-023-00168-9 (PMC10644514; doi:10.1186/s40694-023-00168-9)
Supplement: Supplementary file 14 — Additional file 14: Table S6. List of primers used in this study. [file 40694_2023_168_MOESM14_ESM.docx]

**Table S6. Plasmids used in this study**

| Plasmid | Technical name | Gene | An# (gene) | Gene name | Target sequence | Reference |
| --- | --- | --- | --- | --- | --- | --- |
| pFC332 | pFC332 | - | - | - | - | (70) |
| pTLL108.1 | pTLL108.1 | - | - | - | - | (54) |
| pTLL109.2 | pTLL109.2 | - | - | - | - | (54) |
| pFC332_*mtdA*-sgRNA | pVO1 | NRRL3_04005 | An15g05450 | *mtdA* | GCTGGCAAGACAGCCAGCAG | This study |
| pFC332_*mtdB*-sgRNA | pSJS4 | NRRL3_08606 | An03g02430 | *mtdB* | GAATTTGTCGCAAATCGTGG | This study |
| pFC332_*mpdA*-sgRNA | pVO2 | NRRL3_05796 | An02g05830 | *mpdA* | CGATGAACTTGAGAATGTGG | This study |
| pFC332_*tpsA*-sgRNA | pSJS2 | NRRL3_11571 | An08g10510 | *tpsA* | TCGCGGGTTGACGAAACAAT | This study |
| pFC332_*tpsB*-sgRNA | pSJS3 | NRRL3_04893 | An07g08710 | *tpsB* | TTTGTCGTCCATGAACACCG | This study |
| pFC332_*tpsC*-sgRNA | pSJS4 | NRRL3_00777 | An14g02180 | *tpsC* | TCGCTGAAAAAGGTCGACGG | This study |
| pFC332_KORE1-sgRNA | pTL71.1 | - | - | KORE1 | CCGGCTTATATTGGTACCACTCC | This study |
| pFC332_con10-sgRNA |  | NRRL3_02511 | An01g10790 | *conJ* | AGTGTCGAATATCGCCAAGA | This study |
| pFC332_lea3-sgRNA |  | NRRL3_05684 | An02g07350 | *LEA3-like* | GCCACTGCCCGTCGTGACAA | This study |
| pFC332_dehydrinA-sgRNA |  | NRRL3_01017 | An14g05070 | *dprA* | TGGTCCCCACTCCTCCAACA | This study |
| pFC332_dehydrinB-sgRNA |  | NRRL3_01479 | An13g01110 | *dprB* | CCAGCGCAACCACTGCAACA | This study |
| pFC332_hsf1-sgRNA | pHSF1_1 | NRRL3_07278 | An16g01760 | *hsfA* | ACTGGAACTGGAGAAAACGG | This study |
| pFC332_hsp12-sgRNA | pHSP12_2 | NRRL3_11620 | An06g01610 | *hsp9/12* | CAGCAAGTCCGGTCCCCAGG | This study |
| pFC332_hsp104-sgRNA | pHSP104_2 | NRRL3_02725 | An01g13350 | *hsp104* | GGATCGAGAAGGGCCGTCGG | This study |
