# Supplementary material for: Compatible solutes determine the heat resistance of conidia
Source: Fungal Biol Biotechnol. 2023 Nov 13;10:21. doi: 10.1186/s40694-023-00168-9 (PMC10644514; doi:10.1186/s40694-023-00168-9)
Supplement: Supplementary file 15 — Additional file 15: Table S7. Plasmids used in this study. [file 40694_2023_168_MOESM15_ESM.docx]

**Table S5. List of primers used in this study.**

| Primer | Sequence | | | Purpose |
| --- | --- | --- | --- | --- |
| pTE1_for | CCTTAATTAAACTCCGCCGAACGTACTG | Creation sgRNA on plasmids |  |  |
| pTE1_rev | CCTTAATTAAAAAAGCAAAAAAGGAAGGTACAAAAAAGC | Creation sgRNA on plasmids |  |  |
| tpsA_2_fw | CAGCTGTCGCTTCTCCCATCGTTTTAGAGCTAGAAATAGCAAG | 3’ *tpsA* gRNA (target) |  |  |
| tpsA_2_rv | GATGGGAGAAGCGACAGCTGGACGAGCTTACTCGTTTCG | 5’ *tpsA* gRNA (target) |  |  |
| TS1_tpsA_fw | GTTGTTGCTCGTTAAGTCGGGG | 5' tpsA flank (repair DNA fragment) |  |  |
| TS1_tpsA_rv | ATAAGCCAGTCTCGCCCTTTGTGATTGTTCAACGGCCGAGGATC | 5' tpsA flank (repair DNA fragment) |  |  |
| TS2_tpsA_fw | GATCCTCGGCCGTTGAACAATCACAAAGGGCGAGACTGGCTTAT | 3' tpsA flank (repair DNA fragment) |  |  |
| TS2_tpsA_rv | GCCGGAACTACTCTTGTCCCTT | 3' tpsA flank (repair DNA fragment) |  |  |
| DIAG_tpsA_5'_fw | TTGGTCTTGTAGGGGTAGCTGC | Diagnostic PCR tpsA deletion |  |  |
| DIAG_tpsA_3'_rv | GGTGGTTTTTACTGCTGGGGTG | Diagnostic PCR tpsA deletion |  |  |
| tpsB_fw | TTTGTCGTCCATGAACACCGGTTTTAGAGCTAGAAATAGCAAG | 3’ tpsB gRNA (target) |  |  |
| tpsB_rv | CGGTGTTCATGGACGACAAAGACGAGCTTACTCGTTTCG | 5’ tpsB gRNA (target) |  |  |
| TS1_tpsB_fw | CCATCTGTCTGCCTGTCCTTCA | 5' tpsB flank (repair DNA fragment) |  |  |
| TS1_tpsB_rv | CTCCTTTCGCTCTGCTCTCCATTCTCTTTGGCGAACACAAGCAC | 5' tpsB flank (repair DNA fragment) |  |  |
| TS2_tpsB_fw | GTGCTTGTGTTCGCCAAAGAGAATGGAGAGCAGAGCGAAAGGAG | 3' tpsB flank (repair DNA fragment) |  |  |
| TS2_tpsB_rv | TAGACACCCGAACCAGCAGATG | 3' tpsB flank (repair DNA fragment) |  |  |
| DIAG_tpsB_5'_fw | CAACCGCAACCGCTACTACTTC | Diagnostic PCR tpsB deletion |  |  |
| DIAG_tpsB_3'_rv | CCGGACCAAGGGATGCTAAAGA | Diagnostic PCR tpsB deletion |  |  |
| tpsC_2_fw | TCGCTGAAAAAGGTCGACGGGTTTTAGAGCTAGAAATAGCAAG | 3’ *tpsC* gRNA (target) |  |  |
| tpsC_2_rv | CCGTCGACCTTTTTCAGCGAGACGAGCTTACTCGTTTCG | 5’ *tpsC* gRNA (target) |  |  |
| TS1_tpsC_fw | TGCTCGAGTCTGAGTCTGAAGC | 5' tpsC flank (repair DNA fragment) |  |  |
| TS1_tpsC_rv | GCAGCTCGAAGCATTGCAATTGAGAGACCGTTGGAAGGCTGAAC | 5' tpsC flank (repair DNA fragment) |  |  |
| TS2_tpsC_fw | GTTCAGCCTTCCAACGGTCTCTCAATTGCAATGCTTCGAGCTGC | 3' tpsC flank (repair DNA fragment) |  |  |
| TS2_tpsC_rv | AGCTGGAAGGCGATTGTAGGTT | 3' tpsC flank (repair DNA fragment) |  |  |
| DIAG_tpsC_5'_fw | AATGAATGTGTGTGGGTGCTGC | Diagnostic PCR tpsC deletion |  |  |
| DIAG_tpsC_3'_rv | AAACTGGGAGCGATGCATGAAC | Diagnostic PCR tpsC deletion |  |  |
| mtdA_fw | GCTGGCAAGACAGCCAGCAGGTTTTAGAGCTAGAAATAGCAAG | 3’ mtdA gRNA (target) |  |  |
| mtdA_rv | CTGCTGGCTGTCTTGCCAGCGACGAGCTTACTCGTTTCG | 5’ mtdA gRNA (target) |  |  |
| GOI5_mtdA_fw | CGGTTTGTTTCGGTCTTACGGG | 5' mtdA flank (repair DNA fragment) |  |  |
| XXGOI5_mtdA_rv | GGAGTGGTACCAATATAAGCCGGCGCGGTCGGATAGAAAATAATGT | 5' mtdA flank (repair DNA fragment) |  |  |
| XXGOI3_mtdA_fw | CCGGCTTATATTGGTACCACTCCTGATTGAGGTAGAGATGAGTTTGGT | 3' mtdA flank (repair DNA fragment) |  |  |
| GOI3_mtdA_rv | CTGCAACGTCACTTAGTGGCTG | 3' mtdA flank (repair DNA fragment) |  |  |
| DIAG_mtdA_3'_rv | GCATGCTTGACGTACGGATTGT | Diagnostic PCR mtdA deletion |  |  |
| DIAG_mtdA_5'_fw | CCCCTTGATTTCTCTCCAGCCA | Diagnostic PCR mtdA deletion |  |  |
| mtdB1_fw | GAATTTGTCGCAAATCGTGGGTTTTAGAGCTAGAAATAGCAAG | 3’ mtdB gRNA (target) |  |  |
| mtdB1_rv | CCACGATTTGCGACAAATTCGACGAGCTTACTCGTTTCG | 5’ mtdB gRNA (target) |  |  |
| 5_mtdB_fw | ATCAAGGGATGGAAGGGGTTGG | 5' mtdB flank (repair DNA fragment) |  |  |
| 5_ mtdB _rv | GGAGTGGTACCAATATAAGCCGGGCGGTGTAATTTACCTCTTTGTCGG | 5' mtdB flank (repair DNA fragment) |  |  |
| 3_ mtdB _fw | CCGGCTTATATTGGTACCACTCCTGGGAGGATGAAGGAGGAAGGA | 3' mtdB flank (repair DNA fragment) |  |  |
| 3_ mtdB _rv | AGGTGGCACATGTTCGGTATCA | 3' mtdB flank (repair DNA fragment) |  |  |
| DIAG_MtdB_3'_rv | CGACCAGATCCTCGAAGGGCCA | Diagnostic PCR mtdB deletion |  |  |
| DIAG_MtdB_5'_fw | CTTGCGGAATTTGCGTGGCCAC | Diagnostic PCR mtdB deletion |  |  |
| mpdA_fw | CGATGAACTTGAGAATGTGGGTTTTAGAGCTAGAAATAGC | 3’ mpdA gRNA (target) |  |  |
| mpdA_rv | CCACATTCTCAAGTTCATCGGACGAGCTTACTCGTTTCGT | 5’ mpdA gRNA (target) |  |  |
| GOI5_mpdA_fw | TAGTCGCGAGGGAGTCAAGTTG | 5' mpdA flank (repair DNA fragment) |  |  |
| NEW_GOI5_mpdA_rv | GGAGTGGTACCAATATAAGCCGGATTCCGAGTCGATCACCTGCAT | 5' mpdA flank (repair DNA fragment) |  |  |
| XXGOI3_mpdA_fw | CCGGCTTATATTGGTACCACTCCAGTGGAAGTCTGATAGTAGAAGGGA | 3' mpdA flank (repair DNA fragment) |  |  |
| GOI3_mpdA_rv | TTTGGATTGGCTTGGATTGGGC | 3' mpdA flank (repair DNA fragment) |  |  |
| DIAG_mpdA_3'_rv | AATCAACCGGGACCATGACTGT | Diagnostic PCR mpdA deletion |  |  |
| DIAG_mpdA_5'_fw | CCGACATGGTGATTGCGTCTTC | Diagnostic PCR mpdA deletion |  |  |
| tpsA_KORE2_fw | CAAGAATTACATACCTATGAAGGACAAAGGGCGAGACTGGCTT | Complementation with different KORE (no repair) for SJS156 |  |  |
| tpsA_KORE2_rv | CCTTCATAGGTATGTAATTCTTGGATTGTTCAACGGCCGAGGA | Complementation with different KORE (no repair) for SJS156 |  |  |
| tpsB_KORE3_fw | TTTGTTCACAGTCCTCATTAAGGATGGAGAGCAGAGCGAAAGG | Complementation with different KORE (no repair) for SJS156 |  |  |
| tpsB_KORE3_rv | CCTTAATGAGGACTGTGAACAAATGACTGCAGCTTTTTCCTTG | Complementation with different KORE (no repair) for SJS156 |  |  |
| tpsC_KORE4_fw | AACACCGTTTACCCCCTTAAGGGCAATTGCAATGCTTCGAGCT | Complementation with different KORE (no repair) for SJS156 |  |  |
| tpsC_KORE4_rv | CCCTTAAGGGGGTAAACGGTGTTAGAGACCGTTGGAAGGCTGA | Complementation with different KORE (no repair) for SJS156 |  |  |
| mpdA_KORE5_fw | TCAGTCTATCCGTTTCTTGACGGAGTGGAAGTCTGATAGTAGA | Complementation with different KORE (no repair) for SJS156 |  |  |
| mpdA_KORE5_rv | CCGTCAAGAAACGGATAGACTGATTGCTACTGTCGCAAACTGT | Complementation with different KORE (no repair) for SJS156 |  |  |
| SJS_compl_tpsC_seq_rv | ACGTATTGCCATTCCGTCGGAG | sequence silent mutations tpsC gene |  |  |
| compl_tpsA_fw | CTCATCATCAATGAACACCGGCACG | Create complementation with 2 silent mutations in tpsA gene |  |  |
| compl_tpsA_rv | CGTGCCGGTGTTCATTGATGATGAG | Create complementation with 2 silent mutations in tpsA gene |  |  |
| seq_tpsA_fw | GCCGATCTCCTCACGCAGCATC | sequence silent mutations tpsA gene |  |  |
| seq_tpsA_rv | CGGTGGCCTGGTCAGTGGACTA | sequence silent mutations tpsA gene |  |  |
| compl_tpsB_fw | CAGTTTATCATCCATGAACACCGGG | Create complementation with 2 silent mutations in tpsB gene |  |  |
| compl_tpsB_rv | CCCGGTGTTCATGGATGATAAACTG | Create complementation with 2 silent mutations in tpsB gene |  |  |
| seq_tpsB_fw | AGACAAGGTCTCCTTCCCGGGC | sequence silent mutations tpsB gene |  |  |
| seq_tpsB_rv | TCCATGTCCTCTGGTGGCCTGG | sequence silent mutations tpsB gene |  |  |
| seq_mtdB_fw | ACCCGCATCGTGTCTCTCACCA | sequence silent mutations mtdB gene |  |  |
| seq_mtdB_rv | GTGACGACAGGCCAGGAGTCCT | sequence silent mutations mtdB gene |  |  |
| seq_mpdA_fw | CTCGTCGTCACCAGGCACGTTC | Create complementation with 2 silent mutations in mpdA gene |  |  |
| seq_mpdA_rv | CTCAGCAAGCCCCCAACAGTGG | Create complementation with 2 silent mutations in mpdA gene |  |  |
| mpdA_comsil_fw | CCAAAGTAGGCCGTTGTAGCATGGCTGGTGTTGACAGTGA | sequence silent mutations mpdA gene |  |  |
| mpdA_comsil_rv | CTACAACGGCCTACTTTGGACACTTCCGGGGCAAGAAGAT | sequence silent mutations mpdA gene |  |  |
| SJS_tpsC_complemenationSilent_fw | GTGACTAGTCCACCACTGGAGAGGGAAGATTCGTATCCCC | Create complementation with 2 silent mutations in tpsC gene |  |  |
| SJS_tpsC_complemenationSilent_rv | CTCCAGTGGTGGACTAGTCACATCTTTATCGGGATTGACT | Create complementation with 2 silent mutations in tpsC gene |  |  |
| compl3_mtdB_rv | GCGAGGGCAGCGTAGAGGAAACCGAAAGTGGTACGGGGGG | Create complementation with 2 silent mutations in mpdA gene |  |  |
| compl2_mtdB_fw | CCCCCCGTACCACTTTCGGTTTCCTCTACGCTGCCCTCGC | Create complementation with 2 silent mutations in mpdA gene |  |  |
| Con10_fw | AGTGTCGAATATCGCCAAGAGTTTTAGAGCTAGAAATAGC | 3’ *conJ* gRNA |  |  |
| Con10_rv | TCTTGGCGATATTCGACACTGACGAGCTTACTCGTTTCGT | 5’ *conJ* gRNA |  |  |
| 5_Con10_fw | CCCTGCCATGTAAGTTCCCGCG | 5' *conJ* flank (repair DNA fragment) |  |  |
| 5_Con10_KORE_rv | GGAGTGGTACCAATATAAGCCGGTGATTCTGATCCAATTCCAAACCTCA | 5' *conJ* flank (repair DNA fragment) |  |  |
| 3_Con10_KORE_fw | CCGGCTTATATTGGTACCACTCCTTGAGTGAAGGTACCGCTGGGA | 3' *conJ* flank (repair DNA fragment) |  |  |
| 3_Con10_rv | CGTCGAGTTGAAGCGACCGGAA | 3' *conJ* flank (repair DNA fragment) |  |  |
| DIAG_Con10_5_fw | TAGCCTAGGCTCCCCTTCCCCA | Diagnostic PCR *conJ* deletion |  |  |
| DIAG_Con10_3_rv | ACGCTGCCGCTTACTGTAGCAC | Diagnostic PCR *conJ* deletion |  |  |
| Lea3_fw | GCCACTGCCCGTCGTGACAAGTTTTAGAGCTAGAAATAGC | 3’ *LEA3-like* gRNA |  |  |
| Lea3_rv | TTGTCACGACGGGCAGTGGCGACGAGCTTACTCGTTTCGT | 5’ *LEA3-like* gRNA |  |  |
| 5_Lea3_fw | GGCAGTTGGACTGGGTTTGGGG | 5' *LEA3-like* flank (repair DNA fragment) |  |  |
| 5_Lea3_KORE_rv | GGAGTGGTACCAATATAAGCCGGTCAAGTTGATGGGATTGAGGATGGA | 5' *LEA3-like* flank (repair DNA fragment) |  |  |
| 3_Lea3_KORE_fw | CCGGCTTATATTGGTACCACTCCGCACGCTTGACGACCTGCATGA | 3' *LEA3-like* flank (repair DNA fragment) |  |  |
| 3_Lea3_rv | CCCGGACACTGGCAATTCCGTC | 3' *LEA3-like* flank (repair DNA fragment) |  |  |
| DIAG_Lea3_5_fw | TCACCGACCAGGGGAAGGATGC | Diagnostic PCR *LEA3-like* deletion |  |  |
| DIAG_Lea3_3_rv | TGGAGACGATGGGTCCGCATGA | Diagnostic PCR *LEA3-like* deletion |  |  |
| DehydrinA_fw | TGGTCCCCACTCCTCCAACAGTTTTAGAGCTAGAAATAGC | 3’ *dprA* gRNA |  |  |
| DehydrinA_rv | TGTTGGAGGAGTGGGGACCAGACGAGCTTACTCGTTTCGT | 5’ *dprA* gRNA |  |  |
| 5_DehydrinA_fw | ACCCCAGACTTGGACTCGAGGC | 5' *dprA* flank (repair DNA fragment) |  |  |
| 5_DehydrinA_KORE_rv | GGAGTGGTACCAATATAAGCCGGTGGGCAATTGTATGTGTGTTTGGT | 5' *dprA* flank (repair DNA fragment) |  |  |
| 3_DehydrinA_KORE_fw | CCGGCTTATATTGGTACCACTCCGCGGGCAAACATAAATGCTTGCGT | 3' *dprA* flank (repair DNA fragment) |  |  |
| 3_DehydrinA_rv | ACGTTCCCGCACACATATGCAT | 3' *dprA* flank (repair DNA fragment) |  |  |
| DIAG_DehydrinA_5_fw | GACATCGACGGCACTGGCTGAG | Diagnostic PCR *dprA* deletion |  |  |
| DIAG_DehydrinA_3_rv | CGGAAGGGCTGTTCAACCCACC | Diagnostic PCR *dprA* deletion |  |  |
| DehydrinB_fw | CCAGCGCAACCACTGCAACAGTTTTAGAGCTAGAAATAGC | 3’ *dprB* gRNA |  |  |
| DehydrinB_rv | TGTTGCAGTGGTTGCGCTGGGACGAGCTTACTCGTTTCGT | 5’ *dprB* gRNA |  |  |
| 5_DehydrinB_fw | CCGCAATCCACACTAGGCCGTC | 5' *dprB* flank (repair DNA fragment) |  |  |
| 5_DehydrinB_KORE_rv | GGAGTGGTACCAATATAAGCCGGAGGTAGTATCCATTCCCCACCGT | 5' *dprB* flank (repair DNA fragment) |  |  |
| 3_DehydrinB_KORE_fw | CCGGCTTATATTGGTACCACTCCCGCTATGGGAATGAACCCCGCC | 3' *dprB* flank (repair DNA fragment) |  |  |
| 3_DehydrinB_rv | GAAGATGGAGCACCTCAGGCGC | 3' *dprB* flank (repair DNA fragment) |  |  |
| DIAG_DehydrinB_5_fw | GGCGATCGTGGTGCTCTTGAGG | Diagnostic PCR *dprB* deletion |  |  |
| DIAG_DehydrinB_3_rv | AGAGGATTGGGTGCGCTGGAGT | Diagnostic PCR *dprB* deletion |  |  |
| HSF1_fw_1 | ACTGGAACTGGAGAAAACGGGTTTTAGAGCTAGAAATAGCAAG | PCR target 3’flank |  |  |
| HSF1_rv_1 | CCGTTTTCTCCAGTTCCAGTGACGAGCTTACTCGTTTCG | PCR target 5’ flank |  |  |
| HSP12_fw_2 | CAGCAAGTCCGGTCCCCAGGGTTTTAGAGCTAGAAATAGCAAG | PCR target 3’flank |  |  |
| HSP12_rv_2 | CCTGGGGACCGGACTTGCTGGACGAGCTTACTCGTTTCG | PCR target 5’ flank |  |  |
| HSP104_fw_2 | GGATCGAGAAGGGCCGTCGGGTTTTAGAGCTAGAAATAGCAAG | PCR target 3’flank |  |  |
| HSP104_rv_2 | CCGACGGCCCTTCTCGATCCGACGAGCTTACTCGTTTCG | PCR target 5’ flank |  |  |
| NRRL3-02725-5'-fw | ACGTGCTGGTCAAGTGTATCGA | Donor DNA Hsp104 deletion;  Diagnostic PCR |  |  |
| NRRL3-02725-5'-rv | GGAGTGGTACCAATATAAGCCGGGGTGGTTGATGGGTAGATGGAA | Donor DNA Hsp104 deletion |  |  |
| NRRL3-02725-3'-fw | CCGGCTTATATTGGTACCACTCCGGCGGAATGTGAGGGAAGAATG | Donor DNA Hsp104 deletion |  |  |
| NRRL3-02725-3'-rv | GCTTGAGCATCCCAAGGAGAGA | Donor DNA Hsp104 deletion;  Diagnostic PCR |  |  |
| NRRL3-07278-5'-fw | GAAATCAGGCTTTGGGACAGGC | Donor DNA Hsf1 deletion |  |  |
| NRRL3-07278-5'-rv | GGAGTGGTACCAATATAAGCCGGCGGTCGGTAAAGAGCAAAGACG | Donor DNA Hsf1 deletion |  |  |
| NRRL3-07278-3'-fw | CCGGCTTATATTGGTACCACTCCTGTGTCCGCGGAAGGCAATATA | Donor DNA Hsf1 deletion |  |  |
| NRRL3-07278-3'-rv | TAGGCGATGACACAGACCAAGG | Donor DNA Hsf1 deletion |  |  |
| NRRL3-11620-5'-fw | ATGATACTGCGGATGAGGAGGC | Donor DNA Hsp9/12 deletion;  Diagnostic PCR |  |  |
| NRRL3-11620-5'-rv | GGAGTGGTACCAATATAAGCCGGCGCCACACCCTGATTACAATCG | Donor DNA Hsp9/12 deletion |  |  |
| NRRL3-11620-3'-fw | CCGGCTTATATTGGTACCACTCCCAAGTTGCTCCATGACGTCGAC | Donor DNA Hsp9/12 deletion |  |  |
| NRRL3-11620-3'-rv | TTTGTCTCCCAAGTAGGCCGAG | Donor DNA Hsp9/12 deletion;  Diagnostic PCR |  |  |
