# Supplementary material for: Compatible solutes determine the heat resistance of conidia
Source: Fungal Biol Biotechnol. 2023 Nov 13;10:21. doi: 10.1186/s40694-023-00168-9 (PMC10644514; doi:10.1186/s40694-023-00168-9)
Supplement: Supplementary file 7 — Additional file 7: Fig. S7 The mtdA gene does not have an impact on internal compatible solute composition of conidia. The genes mtdA and mtdB have been deleted in many different backgrounds. Here we give an overview of the effect of the individual mutations on the internal compatible solute profiles of conidia. In all cases, the parental strain without the mtdA or mtdB mutation is given first, followed by the strain in which the mtdA or mtdB gene is additionally knocked-out. a, Here the effect of the mtdA deletion is visualized. When comparing ΔmtdA strains with their respective parental strains, no significant changes in compatible solute profiles were found in any of the knock-out strains where an extra ΔmtdA deletion was made. b, Here the effect of the mtdB deletion is visualized. Initially, in single double or triple knock-out strains the absence of the mtdB gene does not show any significant impact on the compatible solute profiles. However, when deleting mtdB in the ΔmpdA, ΔtpsABC strain, therefore becoming strain ΔmpdA, ΔmtdB, ΔtpsABC, a clear change in compatible solute profile is observed. [file 40694_2023_168_MOESM7_ESM.pdf]

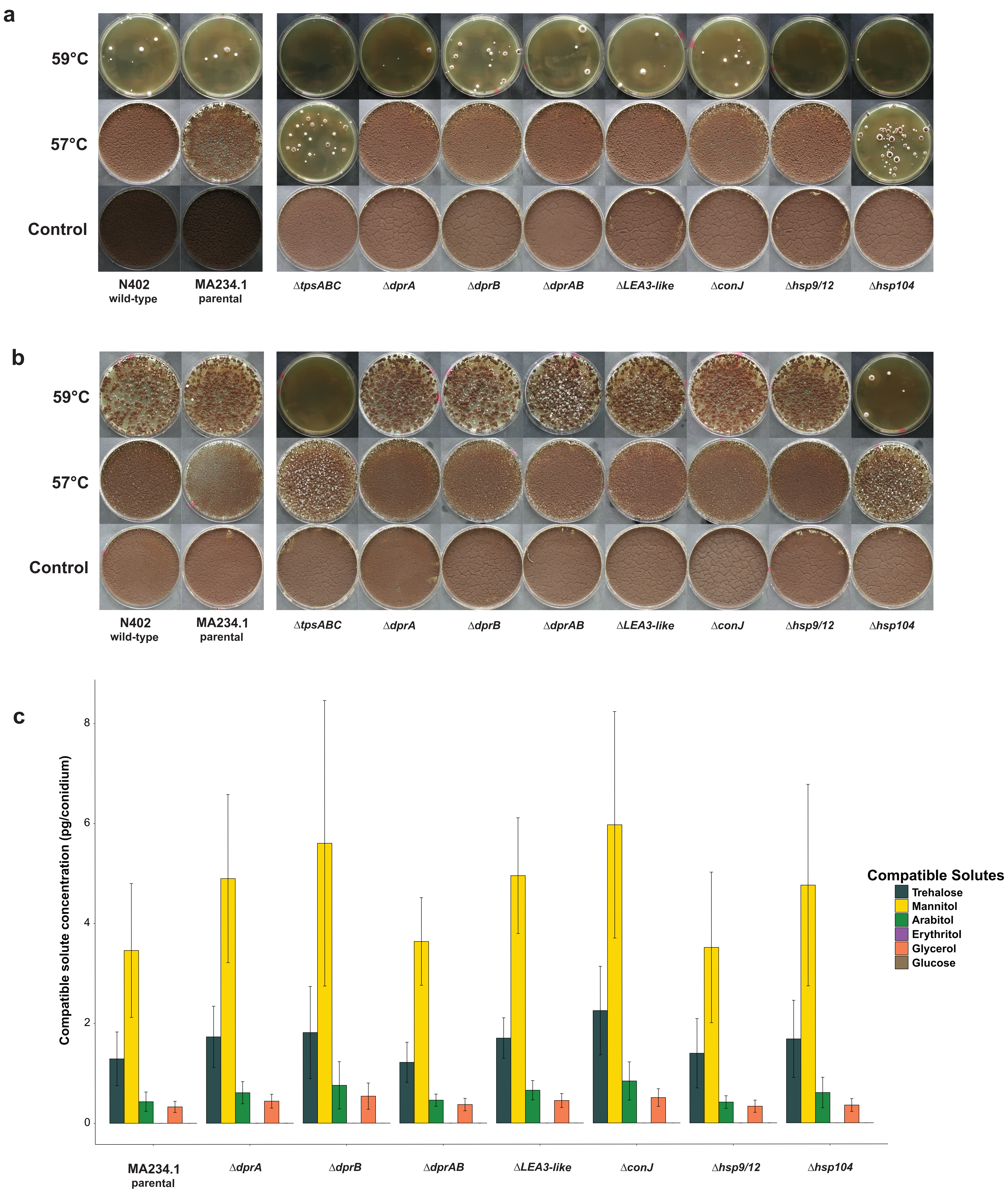

a
59°C
57°C
Control
N402 wild-type
MA234.1 parental
ΔtpsABC
ΔdprA
ΔdprB
ΔdprAB
ΔLEA3-like
ΔconJ
Δhsp9/12
Δhsp104
b
59°C
57°C
Control
N402 wild-type
MA234.1 parental
ΔtpsABC
ΔdprA
ΔdprB
ΔdprAB
ΔLEA3-like
ΔconJ
Δhsp9/12
Δhsp104
c
Compatible solute concentration (pg/conidium)
0
2
4
6
8
MA234.1 parental
ΔdprA
ΔdprB
ΔdprAB
ΔLEA3-like
ΔconJ
Δhsp9/12
Δhsp104
Compatible Solutes
Trehalose
Mannitol
Arabitol
Erythritol
Glycerol
Glucose
